# Retention of ES cell-derived 129S genome drives NLRP1 hypersensitivity and transcriptional deregulation in Nlrp3^tm1Flv^ mice

**DOI:** 10.1038/s41418-024-01379-2

**Published:** 2024-09-17

**Authors:** Felix D. Weiss, Yubell Alvarez, Farhad Shakeri, Anshupa Sahu, Petro Leka, Alesja Dernst, Jessika Rollheiser, Matilde Vasconcelos, Adriana Geraci, Fraser Duthie, Rainer Stahl, Hye Eun Lee, Anne-Kathrin Gellner, Andreas Buness, Eicke Latz, Felix Meissner

**Affiliations:** 1 Institute of Innate Immunity, Department for Systems Immunology and Proteomics, Medical Faculty, University of Bonn, Bonn, Germany; 2 Institute for Medical Biometry, Informatics and Epidemiology, Medical Faculty, University Hospital Bonn, Bonn, Germany; 3 Institute for Genomic Statistics and Bioinformatics, Medical Faculty, University of Bonn, Bonn, Germany; 4 Institute of Innate Immunity, Department for Innate Immunity & Metaflammation, Medical Faculty, University of Bonn, Bonn, Germany; 5 Department of Psychiatry and Psychotherapy, University Hospital Bonn, Bonn, Germany; 6 Institute of Physiology II, Medical Faculty, University of Bonn, Bonn, Germany; 7 Deutsches Rheuma-Forschungszentrum (DRFZ), an Institute of the Leibniz Association, Berlin, Germany

**Keywords:** Cell death and immune response, Immunogenetics

## Abstract

Immune response genes are highly polymorphic in humans and mice, with heterogeneity amongst loci driving strain-specific host defence responses. The inadvertent retention of polymorphic loci can introduce confounding phenotypes, leading to erroneous conclusions, and impeding scientific advancement. In this study, we employ a combination of RNAseq and variant calling analyses to identify a substantial region of 129S genome, including the highly polymorphic *Nlrp1* locus, proximal to *Nlrp3*, in one of the most commonly used mouse models of NLRP3 deficiency (Nlrp3^tm1Flv^). We show that the presence of the Nlrp1^129S^ locus leads to an increase in NLRP1B protein expression, and a sensitising of Nlrp3^tm1Flv^ macrophages to NLRP1 inflammasome activation, independent of NLRP3 deficiency. Retention of 129S genome further leads to protein sequence differences and altered gene regulation across multiple cell types, including of the key tissue-resident macrophage marker, TIM4. Using alternative models of NLRP3 deficiency, including a previously undescribed conditional *Nlrp3* allele enabling precise temporal and cell-type specific control over *Nlrp3* deletion, we further show that NLRP3 contributes to Talabostat-driven IL-1β release. Our study also establishes a generic framework to identify functionally relevant SNPs and assess genomic contamination in transgenic mice using RNAseq data. This allows for unambiguous attribution of phenotypes to the target gene and advances the precision and reliability of research in the field of host defence responses.

## Introduction

Evolutionary pressure results in the emergence of gene paralogs and polymorphisms that shape protein function and regulation. Due to strong selection pressure, immune genes are disproportionately hyperpolymorphic across inbred mouse strains [1], resulting in strain-specific host defence mechanisms, subsequently maintained through generations of inbreeding.

The retention of embryonic stem cell (ESC)-derived genetic material in transgenic mice, especially of polymorphic immune loci, can lead to confounding phenotypes independent of the target gene [2–6], as well as the identification of novel immune defence mechanisms [7].

Inflammasomes are key innate immune signalling hubs that when activated induce a lytic form of cell death known as pyroptosis, and the release of the inflammatory cytokines IL-1β and IL-18. While the inflammasome protein NLRP3 is activated by danger signals including viral infection, potassium efflux and excessive extracellular ATP, the murine NLRP1 inflammasome can be activated by anthrax lethal toxin, *Toxoplasma gondii* (*T. gondii*) infection, and inhibition of dipeptidyl proteases (DPP) 8/9. Whether an endogenous murine NLRP1 activator exists remains unknown.

While, NLRP3 is largely conserved across inbred laboratory mouse strains, the neighbouring *Nlrp1* locus is highly variable [8, 9]. There are five *Nlrp1* genes of which, *Nlrp1a*, *Nlrp1b* and *Nlrp1c-ps* are present in C57B6 strains, and *Nlrp1b* in 129S strains [9]. Furthermore, five distinct *Nlrp1b* alleles, distributed across 14 laboratory mouse strains, display differential sensitivities to a diverse range of stimuli [8], and may be subject to alternative methods of regulation.

NLRP3 has been implicated in a large number of diseases. Murine models of NLRP3 deficiency have shown NLRP3’s causal contribution to the pathogenesis of gout [10] and atherosclerosis [11], through activation by uric acid crystals and cholesterol crystals respectively, however, its mechanistic contribution to diseases including multiple sclerosis [12, 13], Alzheimer’s disease [14, 15], and diet-induced inflammation [16] remains to be clarified. Previous research has shown beneficial effects on disease phenotypes in *Nlrp3*^−/−^ mice even in the absence of clear evidence for NLRP3 inflammasome activation in vivo [13–19], and with limited pharmacological validation. This suggests a potential inflammasome-independent role for NLRP3, or an animal model effect independent of NLRP3 deficiency.

Our study identifies a substantial region of 129S ESC-derived genome in a frequently used model of *Nlrp3* deficiency (Nlrp3^tm1Flv^ [20]). The ~40 Mb region on chromosome 11 contains several hundred genes critical for cell function and identification, and key immune genes, including the *Nlrp1* locus. We show that the presence of alternative Nlrp1 loci, and strain-specific transcriptional and translational regulation of NLRP1 proteins, results in differential NLRP1 inflammasome responses. Differences in gene transcription and protein expression, as well as protein-coding sequences identified in this study, impact innate immune responses independently of the loss of NLRP3 expression, and need to be considered when ascribing mechanistic phenotypes in Nlrp3^tm1Flv^ mice. Validation of a novel inducible *Nlrp3* allele allowing for temporal and cell-type specific control of *Nlrp3* deletion will provide greater clarity on the mechanistic contribution of NLRP3 to disease pathology, in the absence of confounding effects. Finally, our analytical strategy to identify coding variants in relevant expressed genes is applicable to historic and newly generated datasets, enabling a straight-forward analysis of coding variants and genetic heterogeneity in transgenic mice currently considered congenic.

## Results

### Nlrp3^tm1Flv^ mouse macrophages display increased sensitivity to NLRP1 activation by Talabostat

**Fig. 1:**
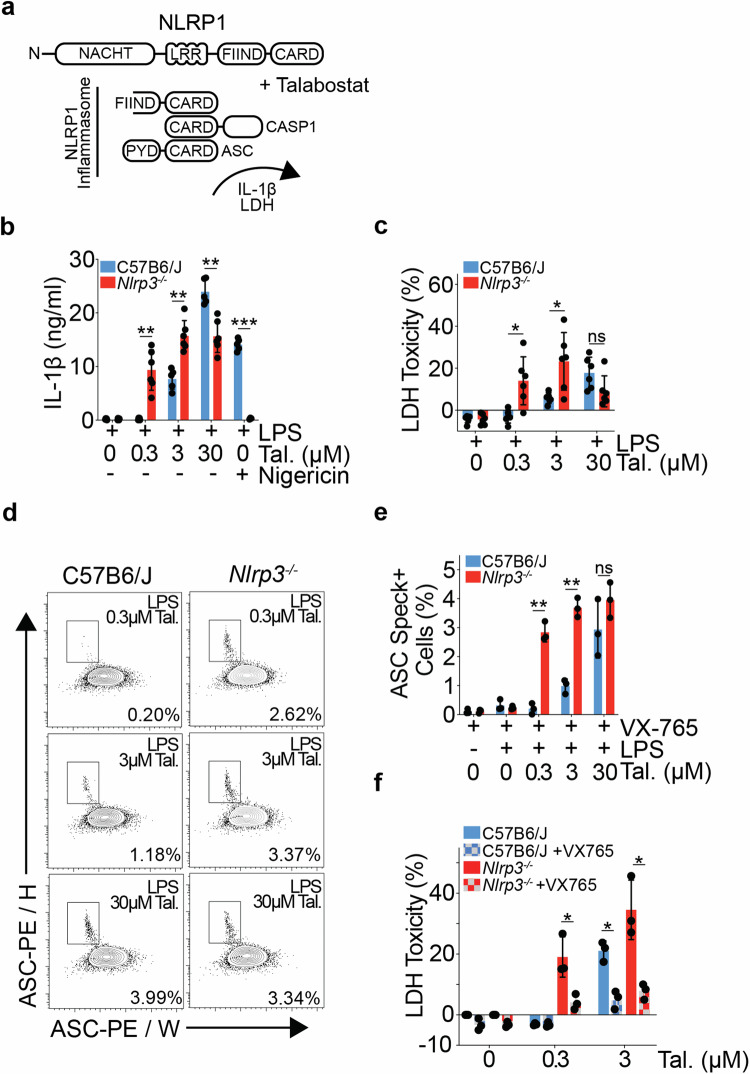
Nlrp3^tm1Flv^ mouse macrophages are hypersensitive to NLRP1 inflammasome activation by Talabostat. **a** Schematic showing murine NLRP1 inflammasome formation in response to Talabostat treatment. **b** IL-1β release from C57B6/J and *Nlrp3*^−/−^ mouse BMDMs stimulated with LPS (10 ng/ml) and Talabostat (0.3, 3 or 30 μM) for 24 h, or primed with LPS (10 ng/ml) for 3 h and then stimulated with Nigericin (8 μM) for 90 min (C57B6/J, *n* = 5, *Nlrp3*^−/−^, *n* = 6). **c** LDH release, relative to untreated total lysis controls, measured from C57B6/J and *Nlrp3*^−/−^ mouse BMDMs stimulated with LPS (10 ng/ml) and Talabostat (0.3, 3 or 30 μM) for 24 h (C57B6/J, *n* = 6, *Nlrp3*^−/−^, *n* = 6). **d** Representative flow cytometry plots showing identification of ASC speck positive cells in C57B6/J and *Nlrp3*^−/−^ mouse BMDMs, pre-gated on live single cells, stimulated with LPS (10 ng/ml) and Talabostat (0.3, 3 or 30 μM) for 16 h, in the presence of VX-765 (50 μM). Percentages represent % of ASC Speck+ cells in the representative plot. **e** Bar plot showing % of ASC speck positive BMDMs from C57B6/J and *Nlrp3*^−/−^ mice stimulated with LPS (10 ng/ml) and Talabostat (0.3, 3 or 30 μM) for 16 h, in the presence of VX-765 (50 μM, C57B6/J, *n* = 3, Nlrp3KO^129ES^, *n* = 3). **f** LDH release, relative to total lysis controls, measured from C57B6/J and *Nlrp3*^−/−^ mouse BMDMs stimulated with Talabostat (0.3 or 3 μM) in the presence or absence of VX-765 (50 μM) for 24 h (C57B6/J, *n* = 3, *Nlrp3*^−/−^, *n* = 3). **b**–**f** All *P* values were calculated using multiple unpaired parametric *t*-tests. FDR (*q*) was calculated using Benjamini–Hochberg correction. *q < 0.05, **q < 0.01, ***q < 0.001, ****q < 0.0001, ns not significant (*q* > 0.05). Error bars represent standard deviation.

DPP8/9 inhibition by Talabostat activates the NLRP1 inflammasome (Fig. 1a [21–25]). We compared the dose-dependent activation of NLRP1 by Talabostat in bone marrow-derived macrophages (BMDMs), generated from C57B6/J and *Nlrp3*^−/−^ (Nlrp3^tm1Flv^) mice, backcrossed >10 generations to C57B6/J and defined by Charles River as congenic.

*Nlrp3*^−/−^ versus C57B6/J BMDMs showed a significant increase in IL-1β secretion at low doses of Talabostat stimulation (Fig. 1b). Consistent with a lower threshold of NLRP1 inflammasome formation, the supernatant LDH levels—indicative of cell membrane rupture by pyroptotic cell death—were also significantly higher in *Nlrp3*^−/−^ BMDMs (Fig. 1c). These data show that *Nlrp3*^−/−^ BMDMs are hypersensitive to DPP8/9 inhibition induced IL-1β release and pyroptosis compared to C57B6/J controls.

Activation of the NLRP1 inflammasome leads to ASC speck formation, where Caspase-1 is recruited and activated. The frequency of ASC speck positive cells was significantly higher at lower doses of Talabostat treatment in *Nlrp3*^−/−^ BMDMs (Fig. 1d, e). These results mirror those observed by IL-1β and LDH release, and show a lower threshold for inflammasome assembly upon NLRP1 activation in *Nlrp3*^−/−^ versus C57B6/J BMDMs. Caspase-1 inhibition by VX-765 significantly rescued cell death, as measured by LDH, in both genotypes (Fig. 1f), showing that increased IL-1β and LDH release is due to the formation of a functional inflammasome.

### Transcriptional deregulation in Nlrp3^tm1Flv^ mouse macrophages is restricted to genes located on chromosome 11

**Fig. 2:**
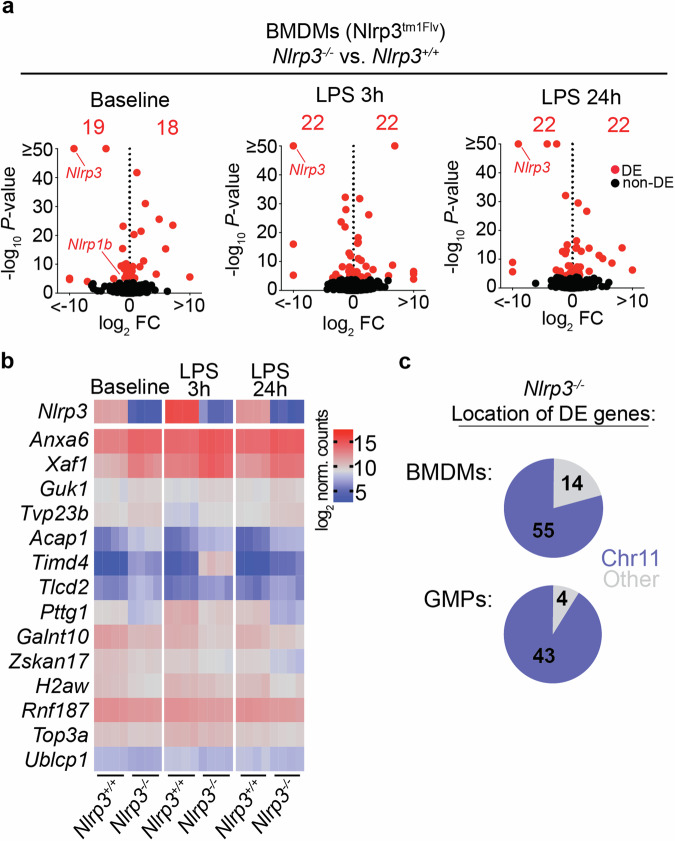
Differentially expressed genes in Nlrp3^tm1Flv^ mice are predominantly located on chromosome 11. **a** Volcano plots of gene expression fold-change versus *P* value in RNAseq of Nlrp3^tm1Flv^ *Nlrp3*^+/+^ and *Nlrp3*^−/−^ BMDMs. Total number of differentially expressed (DE, adj. *P* < 0.05, Benjamini–Hochberg adjusted) genes, and individual DE genes, are shown in red. Left: comparison at baseline. Middle: comparison after 3 h of LPS (10 ng/ml) stimulation. Right: comparison after 24 h of LPS (10 ng/ml) stimulation. **b** Heatmap of log2 normalised counts of genes commonly differentially expressed (adj *P* < 0.05, Benjamini–Hochberg adjusted) in all three comparisons above. **c** Pie charts showing the number of differentially expressed (DE) genes (adj. *P* < 0.05) in BMDMs and GMPs between Nlrp3^tm1Flv^ *Nlrp3*^+/+^ and *Nlrp3*^−/−^ mice, and whether they are located on chromosome 11 (blue) or an alternative chromosome (grey).

In order to determine whether increased sensitivity to NLRP1 activation by Talabostat was due to changes in gene regulation of NLRP1-associated genes, we performed RNA sequencing (RNAseq) of mature polyA RNA from *Nlrp3*^−/−^ and *Nlrp3*^+/+^ (Nlrp3^tm1Flv^) litter-mate controls (Fig. 2a and Supplementary Fig. 1).

Gene expression analysis revealed significant changes between genotypes at baseline and after LPS stimulation, with *Nlrp1b* showing a small but statistically significant reduction in expression in *Nlrp3*^−/−^ BMDMs at baseline, which was not maintained following LPS stimulation (Fig. 2a and Supplementary Table 1).

Additional deregulated genes showed no statistically significant shared functional enrichment as determined by Gene Ontology analysis of biological process or molecular function (adj. *P* < 0.05), and included genes related to annexins (*Anxa6*), apoptosis regulation (*Xaf1*), cell cycle (*Pttg1*) and histones (*H2aw*) among others (Fig. 2b).

Gene expression changes were also identified in FACS-isolated bone marrow resident granulocyte–monocyte progenitors (GMPs, Supplementary Fig. 2a–d and Supplementary Table 2). Despite the limited number of differentially expressed genes, there was highly significant overlap between the two cell types (48% of DE genes in GMPs, 38% of DE genes in BMDMs), including *Xaf1*, *Anxa6, Pttg1*, *H2aw* and *Nlrp1b* (Supplementary Fig. 2d, e).

Strikingly, the majority of significantly differentially expressed genes in both BMDMs and GMPs, including *Nlrp1b*, were located on chromosome 11, which is also home to *Nlrp3* (Fig. 2c).

### Nlrp3^tm1Flv^ mice contain a substantial region of 129S genome proximal to *Nlrp3*, including the highly polymorphic *Nlrp1* locus

Nlrp3^tm1Flv^ mice were originally generated in 129SvEvBRD Lex1 ESCs before being backcrossed >10 generations to C57B6J using speed congenics and defined as congenic by Charles River. Multiple transgenic immune receptor and effector knock-out mouse lines generated in 129 ES cells have been found to contain regions of 129S genome proximal to the target gene despite extensive backcrossing, often confounding results [2–6].

Given that the *Nlrp1* locus is proximal to *Nlrp3* on chromosome 11, this raises the possibility that the observed hypersensitivity in Nlrp3^tm1Flv^ BMDMs to NLRP1 activation could be due to the unintended retention of 129S genome.

**Fig. 3:**
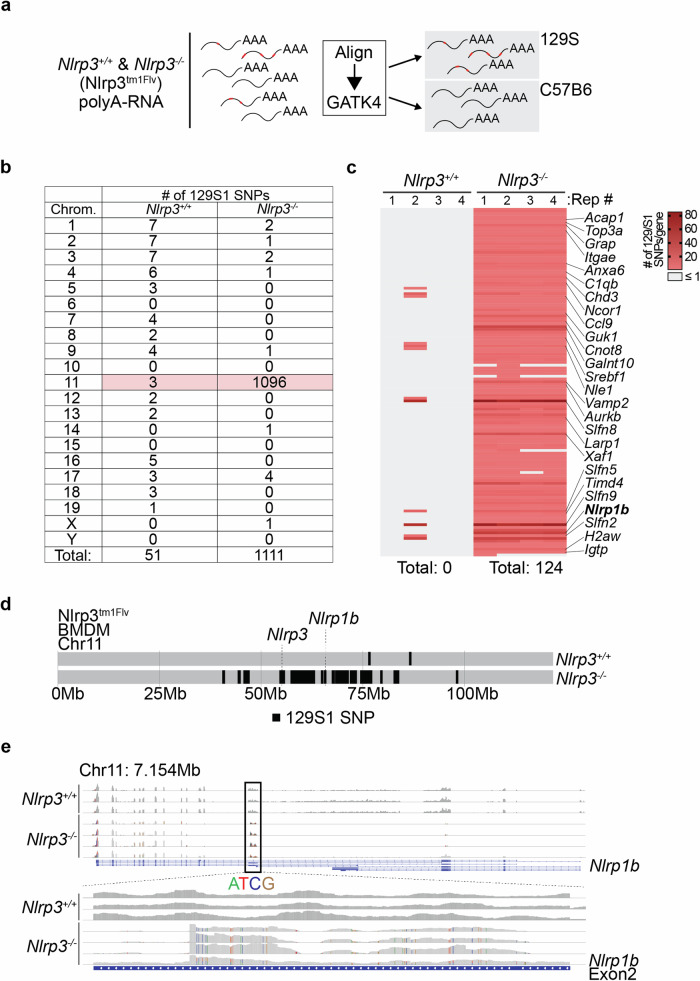
Nlrp3^tm1Flv^ mice contain a ~40 Mb region of 129S chromosome 11, including the Nlrp1^129S^ locus. **a** Schematic representation of variant calling analysis from polyA-RNAseq to identify mouse strain of origin for expressed transcripts. **b** Table showing the number of identified 129S1 SNPs in at least three out of four replicates in both *Nlrp3*^+/+^ and *Nlrp3*^−^^/−^ BMDMs. Values for chromosome 11 are highlighted in red. **c** Heatmap showing number of 129S1 SNPs in genes located on chromosome 11 from both *Nlrp3*^+/+^ and *Nlrp3*^−/−^ BMDMs. Right: subset of genes identified as containing ≥2 129S1 SNPs in at least three out of four biological replicates. Bottom: total number of genes containing ≥2 129S1 SNPs in at least three out of four biological replicates. **d** Line plot showing the location of *Nlrp3* and *Nlrp1b*, and every identified SNP mapping to the 129S1 genome compared to the reference genome in RNAseq data from *Nlrp3*^+/+^ and *Nlrp3*^−/−^ BMDMs on chromosome 11. Based on presence in three out of four biological replicates in each genotype. **e** Snapshot from the Integrative Genomics Viewer (IGV) showing RNAseq reads of *Nlrp1b* from *Nlrp3*^+/+^ and *Nlrp3*^−/−^ BMDMs at baseline. Top: read coverage over the whole *Nlrp1b* gene (expression range: 0–300). Bottom: zoom view of reads at exon 2 of *Nlrp1b* (expression range: 0–100). SNPs are highlighted in separate colours (adenine (A) = green, thymine (T) = red, cytosine (C) = blue, guanine (G) = brown).

In order to determine whether and to what degree Nlrp3^tm1Flv^ mice retained 129S genome, and which genes expressed in macrophages are of 129S origin, we performed variant calling analysis on polyA-RNAseq reads from *Nlrp3*^−/−^ and *Nlrp3*^+/+^ litter-mate control macrophages (Fig. 3a).

In *Nlrp3*^−/−^ BMDMs, we identified 1111 SNPs matching the 129S1 genome in at least three out of four biological replicates, with 1096 SNPs located on chromosome 11 (Fig. 3b and Supplementary Fig. 3a). By contrast, only 51 such SNPs matching the 129S1 genome were found in RNAseq data from *Nlrp3*^+/+^ BMDMs, with three SNPs on chromosome 11 (Fig. 3b and Supplementary Fig. 3b).

As variant calling was performed using RNAseq reads generated from mature polyA RNA, all SNPs identified are located in the coding regions of mRNA from genes expressed in BMDMs. In total, 124 genes located on chromosome 11 in *Nlrp3*^−^^/−^ BMDMs contained ≥2 129S1 SNPs, in at least three out of four biological replicates (Fig. 3c and Supplementary Table 3). No genes matching to 129S1 were identified on chromosome 11 of *Nlrp3*^+/+^ BMDMs using the same criteria (Fig. 3c and Supplementary Table 4). These genes spanned a region of ~40 Mb around *Nlrp3* (Fig. 3d), home to a total of 715 genes, and genes identified as being of 129S origin included those previously identified as significantly deregulated, including *Anxa6*, *Xaf1* and *H2aw* (Fig. 3c). Furthermore, multiple genes critical for immune cell function were found to be of 129S origin, including *C1qbp*, *Ccl9*, *Igtp*, *Timd4*, and critically, *Nlrp1b* (Fig. 3c, e). Similar results were observed in GMPs, where 956 SNPs matching the 129S1 genome were observed, with ≥2 129S1 SNPs within 120 genes (Supplementary Table 5).

We extended our analysis to microglia in order to determine whether expression changes of genes located on chromosome 11 in *Nlrp3*^−^^/−^ mice were not just limited to cultured BMDMs and their precursors. Gene expression analysis of microglia purified from adult mouse brains of Nlrp^3tm1Flv^ *Nlrp3*^−/−^ and *Nlrp3*^+/+^ litter-mate controls also revealed a significant deregulation of multiple genes located on chromosome 11 including those with critical immune cell functions, such as *Ubb*, *Ccl4*, *Ccl3*, *Pttg1*, *Xaf1* and again *Nlrp1b* (Supplementary Fig. 4 and Supplementary Table 6).

These data show that Nlrp3^tm1Flv^ mice contain a substantial region of 129S mouse strain genome on chromosome 11, in the region surrounding *Nlrp3*, as a result of their production in 129S ESCs, despite extensive backcrossing and validation as congenic. The presence of polymorphisms both in coding and regulatory regions can impact gene and protein expression, as well as the ability to accurately quantify transcripts. In such cases, alternative analysis approaches are required using multiple reference genomes to determine gene expression changes [26].

### Nlrp3^tm1Flv^ BMDMs display strain-specific regulation of NLRP1 inflammasome transcripts and proteins

**Fig. 4:**
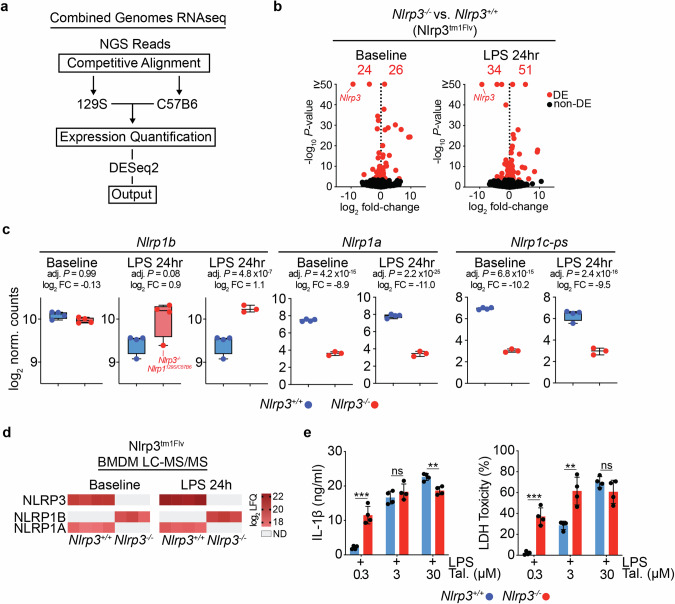
Combined genomes RNAseq and LC–MS/MS analysis reveals differential expression of Nlrp1 genes and proteins in Nlrp3^tm1Flv^ macrophages. **a** Schematic representation of RNAseq analysis using both 129S and the C57B6-based reference genome for gene expression analysis. **b** Volcano plots of gene expression fold-change versus *P* value in RNAseq of Nlrp3^tm1Flv^ *Nlrp3*^+/+^ and *Nlrp3*^−^^/−^ BMDMs using combine genomes RNAseq. Total number of differentially expressed (DE, adj. *P* < 0.05, Benjamini–Hochberg adjusted) genes, and individual DE genes, are shown in red. Left: comparison at baseline. Right: comparison after 24 h of LPS (10 ng/ml) stimulation. **c** Box plots of *Nlrp1a*, *Nlrp1b* and *Nlrp1c-ps* gene expression in *Nlrp3*^−^^/−^ and *Nlrp3*^+/+^ BMDMs at baseline and after 24 h of LPS (10 ng/ml) stimulation (adj. *P*, Benjamini–Hochberg adjusted, FC = fold-change). **d** Heatmap of log_2_ LFQ values of NLRP3, NLRP1B and NLRP1A proteins from LC–MS/MS of *Nlrp3*^+/+^ and *Nlrp3*^−^^/−^ BMDMs at baseline and after 24 h of LPS (10 ng/ml) stimulation. Grey spaces indicate samples where proteins were not detected. **e** IL-1β release and LDH release relative to total lysis controls from Nlrp3^tm1Flv^ litter-mate *Nlrp3*^+/+^ and *Nlrp3*^−/−^ BMDMs stimulated with LPS (10 ng/ml) and Talabostat (0.3, 3 or 30 μM) for 24 h (*Nlrp3*^+/+^, *n* = 4, *Nlrp3*^−/^^−^, *n* = 4). *P* values were calculated using multiple unpaired parametric *t*-tests. FDR (*q*) was calculated using two-stage step-up Benjamini, Krieger and Yekutieli. *q < 0.05, **q < 0.01, ***q < 0.001, ****q < 0.0001, ns not significant (*q* > 0.05). Error bars represent standard deviation.

Given that Nlrp3^tm1Flv^ mice contain a substantial region of 129S genome, and express transcripts known to display polymorphisms to C57B6, including at the *Nlrp1* locus, we aligned transcripts to both the 129S and standard C57B6-based reference genomes to accurately quantify gene expression changes in *Nlrp3*^−/−^ BMDMs (Fig. 4a). This analysis identified 52 and 85 significant differentially expressed genes at baseline and following 24 h of LPS treatment, respectively (Fig. 4b).

The combined genome RNAseq analysis revealed a significant upregulation of *Nlrp1b* in *Nlrp3*^−^^/−^ BMDMs following 24 h of LPS treatment (Fig. 4c and Supplementary Table 7), with the exception of one sample which was identified as *Nlrp1*^*129S/C57B6*^ and subsequently excluded. Consistent with known literature, there was a significant downregulation of *Nlrp1a* and *Nlrp1c-ps* as both genes are not present in the Nlrp1^129S^ locus (Fig. 4c).

Protein expression analysis by liquid chromatography coupled to mass spectrometry (LC–MS/MS) corroborated gene expression data. *Nlrp3*^−^^/−^ BMDMs did not express NLRP3 or NLRP1A, but robustly expressed NLRP1B, whereas *Nlrp3*^+/+^ BMDMs expressed NLRP3 and NLRP1A, while NLRP1B was not detected (Fig. 4d and Supplementary Table 8). *Nlrp1c-ps* is a pseudogene which is not translated and therefore not detected by LC–MS/MS. Finally, Nlrp3^tm1Flv^ litter-mate controls displayed the same hypersensitivity to Talabostat as observed between *Nlrp3*^−/−^ and C57B6J BMDMs (Fig. 4e).

These data show that the increase in sensitivity coincides with the presence of the Nlrp1^129S^ locus and the detection of NLRP1B by LC–MS/MS, and raises the possibility that NLRP1 hypersensitivity in Nlrp3^tm1Flv^ BMDMs is not due to NLRP3 deficiency, but instead to the presence of the Nlrp1^129S^ locus and the differential expression of NLRP1 proteins.

### NLRP1 hypersensitivity is associated with the presence of alternative *Nlrp1* loci, not NLRP3 deficiency

In order to determine whether NLRP1 hypersensitivity observed in Nlrp3^tm1Flv^ BMDMs was due to NLRP3 deficiency or the presence of the Nlrp1^129S^ locus, we analysed NLRP1 inflammasome activation in alternative genetic and pharmacological models of NLRP3 deficiency.

**Fig. 5:**
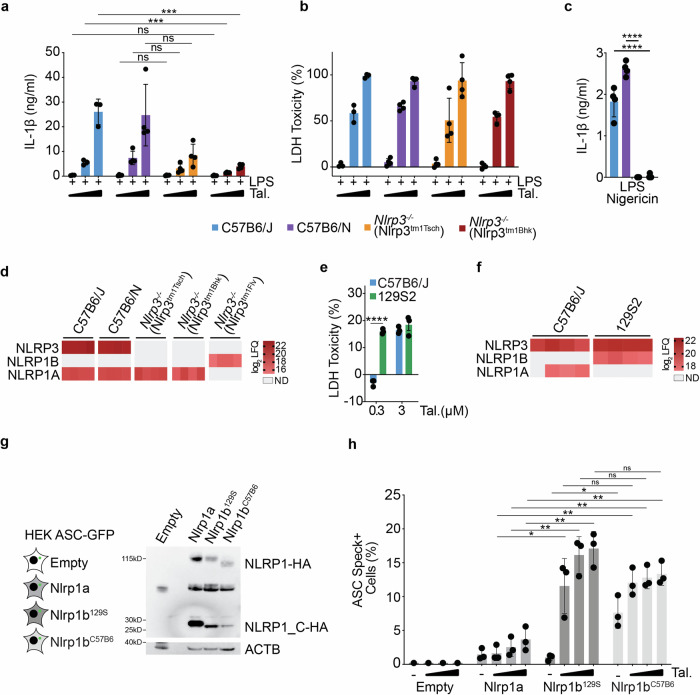
Presence and expression of the NLRP1^129S^ locus, not NLRP3 deficiency, drives hypersensitivity to Talabostat. **a** IL-1β release from C57B6/J, C57B6/N, *Nlrp3*^−^^/^^−^ (Nlrp3^tm1Tsch^) and *Nlrp3*^−^^/−^ (Nlrp3^tm1Bhk^) BMDMs stimulated with LPS (10 ng/ml) and Talabostat (0.3, 3 or 30 μM) for 24 h (C57B6/J, *n* = 3, C57B6/N, Nlrp3^tm1Tsch^ and Nlrp3^tm1Bhk^, *n* = 4). **b** LDH release relative to total lysis controls from C57B6/J, C57B6/N, *Nlrp3*^−^^/−^ (Nlrp3^tm1Tsch^) and *Nlrp3*^−^^/−^ (Nlrp3^tm1Bhk^) BMDMs stimulated with LPS (10 ng/ml) and Talabostat (0.3, 3 or 30 μM) for 24 h (C57B6/J, *n* = 3, C57B6/N, Nlrp3^tm1Tsch^ and Nlrp3^tm1Bhk^, *n* = 4). **c** IL-1β release from C57B6/J, C57B6/N, *Nlrp3*^−^^/−^ (Nlrp3^tm1Tsch^) and *Nlrp3*^−^^/−^ (Nlrp3^tm1Bhk^) BMDMs primed with LPS (10 ng/ml) for 3 h and then stimulated with Nigericin (8 μM) for 90 min (*n* = 4). **d** Heatmap of log_2_ LFQ values of NLRP3, NLRP1B and NLRP1A proteins from C57B6/J, C57B6/N, *Nlrp3*^−^^/−^ (Nlrp3^tm1Tsch^) and *Nlrp3*^−^^/^^−^ (Nlrp3^tm1Bhk^) BMDMs stimulated with LPS (10 ng/ml) for 24 h (*n* = 4). Grey spaces indicate samples where proteins were not detected (ND). **e** LDH release, relative to total lysis controls, from C57B6/J and 129S2 BMDMs after stimulation with Talabostat (0.3 or 3 μM) for 24 h (*n* = 3). **f** Heatmap of log_2_ LFQ values of NLRP3, NLRP1B and NLRP1A proteins from C57B6/J and 129S2 BMDMs. Grey spaces indicate samples where proteins were not detected (ND). **g** Western blot analysis of C-terminal HA tagged NLRP1A, NLRP1B^129S^ and NLRP1B^C57B6^ expression in HEK ASC-GFP cells. **h** Bar plot showing % of ASC speck positive HEK ASC-GFP cells empty or either stably expressing NLRP1A, NLRP1B^129S^ or NLRP1B^C57B6^ stimulated with Talabostat (0.3, 3 or 30 μM) for 16 h (NLRP1A, NLRP1B^129S^, NLRP1B^C57B6^: *n* = 3, Empty: *n* = 1). **a**–**h** *P* values were calculated using multiple unpaired parametric *t*-tests. FDR (*q*) was calculated using Benjamini–Hochberg correction. *q < 0.05, **q < 0.01, ***q < 0.001, ****q < 0.0001, ns not significant (*q* > 0.05). Error bars represent standard deviation.

Two alternative transgenic murine models of constitutive NLRP3 deficiency, Nlrp3^tm1Tsch^ (generated in C57B6 ESCs [10]), and Nlrp3^tm1Bhk^ (generated in 129S ESCs [27], but containing the Nlrp1^C57B6^ locus, Supplementary Fig. 5a) did not display hypersensitivity to Talabostat stimulation compared to C57B6/N or J controls as determined by IL-1β and LDH release (Fig. 5a, b). As expected, they displayed a significant reduction in LPS + Nigericin-dependent IL-1β release (Fig. 5c).

These data were validated through pharmacological inhibition of NLRP3 by MCC950, a highly selective and potent inhibitor of NLRP3, as well as tamoxifen dependent NLRP3 depletion, neither of which displayed a hypersensitive response to Talabostat (Supplementary Fig. 5b–f). Surprisingly, multiple models of NLRP3 deficiency resulted in a significant reduction of IL-1β release in response to high doses of Talabostat (Fig. 5a and Supplementary Fig. 5b, f) suggesting a previously undescribed role for NLRP3 in Talabostat-mediated inflammasome activation.

Consistent with previous observations that NLRP1B protein detection is associated with Talabostat hypersensitivity, LC–MS/MS analysis only detected NLRP1B in Nlrp3^tm1Flv^ BMDMs, and not in C57B6/J, C57B6/N, Nlrp3^tm1Tsch^ or Nlrp3^tm1Bhk^ BMDMs (Fig. 5d and Supplementary Table 9). NLRP1A was detected in all mouse strains containing the Nlrp1^C57B6^ locus (Fig. 5d).

In order to determine whether the Nlrp1^129S^ locus was still capable of inducing Talabostat hypersensitivity in the presence of NLRP3, we analysed the response of wild-type 129S2 and C57B6/J BMDMs. Wild-type 129S2 BMDMs released significantly higher amounts of LDH in response to Talabostat compared to C57B6/J (Fig. 5e), phenocopying the results observed in Nlrp3^tm1Flv^ BMDMs. Analysis of protein expression by LC–MS/MS revealed a familiar pattern of protein expression, where hypersensitive 129S2 BMDMs express NLRP1B, and C57B6 BMDMs express NLRP1A but not NLRP1B (Fig. 5f and Supplementary Table 10). Both BMDM populations expressed NLRP3 (Fig. 5f).

We analysed ASC speck formation in HEK ASC-GFP reporter cells expressing NLRP1A, NLRP1B^C57B6^ or NLRP1B^129S^ to determine the inflammasome forming capacity of different murine NLRP1 proteins (Fig. 5g). HEK reporters expressing NLRP1A showed a significant reduction in ASC speck formation in response to Talabostat, compared to those expressing NLRP1B^C57B6^ or NLRP1B^129S^ (Fig. 5h). There was no significant difference in Talabostat induced ASC speck formation between NLRP1B^C57B6^ and NLRP1B^129S^ expressing HEK reporters (Fig. 5h). As previously reported, NLRP1B^C57B6^ expressing HEK ASC-GFP cells display a significant increase in spontaneous speck formation in the absence of any triggers compared to those expressing NLRP1B^129S^ (Fig. 5h [28]).

We conclude that the hypersensitivity to Talabostat observed in Nlrp3^tm1Flv^ BMDMs is associated with the presence of the Nlrp1^129S^ locus, the increased expression of NLRP1B, and is independent of NLRP3 deficiency or an intrinsic increase in NLRP1B^129S^ sensitivity. We further show that NLRP1A only weakly forms inflammasomes in response to Talabostat. Finally, our data also show that NLRP3 contributes to Talabostat-driven IL-1β maturation or release.

### Nlrp3^tm1Flv^ mice misexpress the canonical macrophage marker TIM4

**Fig. 6:**
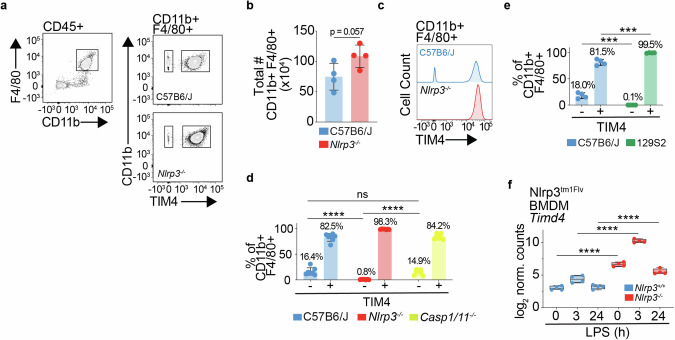
Monocyte-derived peritoneal macrophages from Nlrp3^tm1Flv^ mice overexpress TIM4. **a** Representative flow cytometry plots showing identification of TIM4− and TIM4+ macrophages (F4/80+ CD11b+) in C57B6/J and *Nlrp3*^−/−^ (Nlrp3^tm1Flv^) mouse peritoneum. Cells were pre-gated on single, live and CD45+ populations. **b** Bar plot of the total number of macrophages (CD11b+ F4/80+) in each peritoneum of each mouse (*Nlrp3*^−/−^, *n* = 4; C57B6/J, *n* = 4). *P* value was calculated using an unpaired *t*-test. Error bars represent standard deviation. **c** Representative histogram of distribution of TIM4− and TIM4+ macrophages (F4/80+ CD11b+) in C57B6/J and *Nlrp3*^−^^/^^−^ (Nlrp3^tm1Flv^) mouse peritoneum. **d** Bar plot of TIM4− and TIM4+ macrophages (F4/80+ CD11b+) in C57B6/J, *Nlrp3*^−/−^ (Nlrp3^tm1Flv^) and *Casp1/11*^*−/−*^ mouse peritoneum. Percentages above bars show the mean value for each group. (*Nlrp3*^−^^/−^, *n* = 7; C57B6/J, *n* = 7; *Casp1/11*^−/−^, *n* = 8). Adj. *P* values were calculated using a two-way ANOVA with Turkey’s multiple comparison testing. *adj. *P* < 0.05, **adj. *P* < 0.01, ***adj. *P* < 0.001, ****adj. *P* < 0.0001, ns not significant (adj. *P* > 0.05). Error bars represent standard deviation. **e** Bar plot of TIM4− and TIM4+ macrophages (F4/80+ CD11b+) in C57B6/J and 129S2 mouse peritoneum. Percentages above bars show the mean value for each group (C57B6/J, *n* = 4; 129S2, *n* = 4). *P* values were calculated using multiple unpaired parametric *t*-tests. FDR (*q*) was calculated using Benjamini–Hochberg correction. *q < 0.05, **q < 0.01, ***q < 0.001, ****q < 0.0001, ns not significant (*q* > 0.05). Error bars represent standard deviation. **f** log_2_ normalised counts of *Timd4* from RNAseq of *Nlrp3*^−^^/−^ and *Nlrp3*^+/+^ (Nlrp3^tm1Flv^) BMDMs at baseline and after 3 and 24 h of LPS (10 ng/ml) stimulation (*Nlrp3*^−^^/−^, *n* = 4; *Nlrp3*^+/+^, *n* = 4). *adj. *P* < 0.05, **adj. *P* < 0.01, ***adj. *P* < 0.001, ****adj. *P* < 0.0001, ns not significant. Wald Test, Benjamini–Hochberg corrected.

The peritoneal cavity macrophage population can be broadly separated into two functionally distinct subsets: short-lived monocyte-derived macrophages and long-lived tissue-resident macrophages. Monocyte-derived macrophages are inflammatory and invade the peritoneum during inflammation, while long-lived tissue-resident macrophages regulate tissue homoeostasis. In multiple organs including the peritoneal cavity, the cell surface protein TIM4 is used to distinguish long-lived tissue-resident macrophages (TIM4+) from short-lived monocyte-derived macrophages (TIM4−, Fig. 6a and Supplementary Fig. 6 [29–34]). As such TIM4 is a critical tool in understanding tissue biology and the role of myeloid cells in inflammation.

Analysis of peritoneal macrophage subtypes revealed that, despite having the same total number of macrophages in the peritoneum (Fig. 6b), TIM4− macrophages appeared to be virtually absent from Nlrp3^tm1Flv^ peritoneal cavity (Fig. 6c). Quantification of TIM4+ and TIM4− macrophages as a percentage of total macrophages (CD11b+ F4/80+) revealed a significant decrease in the percentage of TIM4− macrophages in the Nlrp3^tm1Flv^ peritoneal cavity compared to C57B6/J (16.4%–0.8%, adj. *P* < 0.0001, Fig. 6d).

The absence of TIM4− macrophages in Nlrp3^tm1Flv^ was not due to the loss of tonic NLRP3 signalling as *Casp1/11*^−^^/−^ mice showed no significant difference in percent frequency of TIM+ and TIM4− macrophages relative to C57B6/J control mice, and significantly more TIM4− macrophages than Nlrp3^tm1Flv^ mice as a percent of total macrophages (14.9%–0.8%, adj. *P* < 0.0001, Fig. 6d). Strikingly, TIM4− macrophages were also absent from 129S2 mice (Fig. 6e), suggesting that the lack of TIM4− macrophages in Nlrp3^tm1Flv^ mice could be another strain-specific effect caused by the retention of 129S ESC-derived genome.

RNAseq analysis of *Nlrp3*^−^^/−^ (Nlrp3^tm1Flv^) BMDMs, which are monocyte derived, revealed that the TIM4 coding gene *Timd4* is highly significantly upregulated in Nlrp3^−^^/−^ and BMDMs (Fig. 6f). *Timd4* is located on chromosome 11 (Chr11: 46,808,799) in close proximity to *Nlrp3* (Chr11: 59,539,569). Variant analysis of RNAseq data identified three SNPs in *Timd4*, defining it as of 129S origin.

Therefore, monocyte-derived macrophages are not absent from the Nlrp3^tm1Flv^ peritoneum, but instead aberrantly upregulate the normally tissue-resident macrophage restricted marker TIM4 as a result of the presence of 129S genome, confounding cell identification by flow cytometry.

## Discussion

In this study we use conventional and combined genomes RNAseq with variant calling analysis to identify genetic variation in one of the most frequently used mouse models of NLRP3 deficiency. We identify the retention of a ~40 Mb region of 129S ESC-derived genomic material proximal to *Nlrp3*, leading to changes in gene regulation across multiple cell types, peptide sequences, the differential expression of polymorphic proteins, and alterations to the NLRP1 inflammasome response.

Post-transcriptional and -translational regulation of NLR proteins is critical for immune homoeostasis, and the upregulation of inflammasome proteins is a key priming step before activation and pyroptosis. We show that the presence of the Nlrp1^129S^ locus results in the LPS-dependent upregulation of Nlrp1b transcript and protein compared to Nlrp1^C57B6^ mice. Macrophages containing the Nlrp1^129S^ locus, and expressing NLRP1B but not NLRP1A, are hypersensitive to NLRP1 activation by Talabostat, regardless of NLRP3 expression. Multiple alternative murine models of NLRP3 deficiency do not contain Nlrp1^129S^, including conditional alleles, reducing the impact of confounders.

Gain-of-function mutations to inflammasome proteins cause severe auto-inflammatory diseases in humans. Our data shows that NLRP1B^C57B6^, unlike NLRP1B^129S^, can form inflammasomes in the absence of any triggers, likely due to the substantial peptide sequence heterogeneity between the two alleles. This suggests that through evolutionary pressure, the Nlrp1^C57B6^ locus or *Nlrp1b*^*C57B6*^ gene itself have accrued polymorphisms that restrict its expression, potentially through both transcriptional and post-translational mechanisms, protecting mice from spontaneous inflammasome formation. While mice containing the Nlrp1^129S^ locus can therefore tolerate higher expression levels of NLRP1B at baseline, they are subsequently sensitised to NLRP1 inflammasome triggers, as observed in Nlrp3^tm1Flv^ macrophages.

The exclusive detection of NLRP1A and not NLRP1B in mice containing the Nlrp1^C57B6^ locus, and the reduction in NLRP1A sensitivity to stimulation by Talabostat, warrants further mechanistic interrogation. NLRP1A and NLRP1B proteins display a high degree of sequence heterogeneity, and which individual mutations or regions define the differences in inflammasome forming ability remains to be resolved. Furthermore, our work raises additional questions of whether phenotypes previously ascribed to strain-specific sequence differences of NLRP1B, such as macrophage sensitivity to *T. gondii* infection [35], are in fact due to the differential expression of NLRP1A and NLRP1B.

Changes in the expression of immune genes are not limited to the *Nlrp1* locus. The upregulation of *Timd4* in Nlrp3^tm1Flv^ monocyte-derived macrophages results in their misidentification as tissue-resident macrophages due to increase surface expression of TIM4. As a result, TIM4 cannot be used to determine frequencies of monocyte-derived versus tissue-resident macrophages in Nlrp3^tm1Flv^ mice, and heterogenous cell populations are obtained when purifying cells for molecular analysis such as RNAseq or LC–MS/MS, as well as confounding population analyses by FACS. Beyond cell identification, TIM4 is involved in mediating the clearance of apoptotic cells [36–38], regulating cholesterol metabolism [39, 40] and enabling cross-presentation of tumour antigens [41]. Therefore, potential phenotypic changes in Nlrp3^tm1Flv^ macrophages driven by the differential expression of TIM4 should also be considered.

Other immune genes identified as containing 129S SNPs include *Igtp*, an interferon induced gene critical for host defence [42], *C1qbp*, a complement family member that aids the clearance of apoptotic cells [43], *Itgae*, which codes for the dendritic [44] and T cell [45] marker CD103, *Xaf1*, an antagonist of the anti-apoptosis protein XIAP [46] and the chemokine *Ccl9*, amongst others. Further investigations in Nlrp3^tm1Flv^ mice are required to assess the contribution of these differences to immune responses. While unlikely, we also cannot exclude that the presence of contaminating 129S genome directly or indirectly impacts the regulation of the NLRP1 inflammasome, and further work could pursue individual candidates.

Genes required for conserved functions across multiple cell types were also found to contain 129S SNPs including but not limited to genes related to cell cycle (*Pttg1*, *Aurkb*, *Ccng1*), trafficking (*Gosr1*), histones (*H2aw*), metabolism (*Mat2b, Guk1, Srebf1*, *Galnt10*) translation (*Larp1*, *Mm3*) and transcription (*Cnot8, Top3a*, *Chd3*, *Pol2ra*, *Ncor1*, *Mnt*).

Finally, the region around *Nlrp3* that we detect as being of 129S origin contains genes not expressed in macrophage or GMPs, but of importance in functions such as neuronal signalling (*Gabrg2, Gabra1, Gabra6, Gria1*), olfaction (80 olfactory receptor genes), and development (*Wnt3a*), among others. Further variant calling analysis either on DNA sequencing data or RNAseq from relevant cell types would be needed to determine the full scale of 129S contamination and the effect on cell function, which could have significant knock-on effects due to alterations in paracrine signalling.

In transgenic mice, ESC-derived genome retention represents a minority, but potentially functionally important, proportion of the genome. Whole exome sequencing does not inform researchers on which mutated genes are expressed, and to what degree. Our approach to identify genetic heterogeneity in transgenic mice using variant calling analysis on RNAseq data allows for the detection of SNPs in coding regions of functionally relevant expressed genes. Furthermore, it allows for the post hoc analysis of genetic heterogeneity in historical samples that utilised RNAseq.

Our identification of 129S genome in Nlrp3^tm1Flv^ mice, and corresponding changes in gene and protein expression, peptide sequences, and immune response, highlights the careful consideration that should be given to ascribing phenotypes in Nlrp3^tm1Flv^ mice to NLRP3 deficiency. Alternative models of NLRP3 deficiency generated in C57B6 ESCs, for example, those described in this study (Nlrp3^tm1Tsch^, Nlrp3^fl/fl^) as well as Nlrp3^tm1Vmd^ [47] could be used to validate observations and eliminate potential confounders. Indeed, Nlrp3^tm1Tsch^ and Nlrp3^tm1Vmd^ have been used extensively to further the field of NLRP3 biology and its role in disease. Further work in Nlrp3^tm1Flv^ mice should evaluate or consider the full scale of disruption caused by 129S genomic retention, as many other cell types may be altered by genetic differences or paracrine activities from affected cells.

## Materials and methods

### Mice

Mice were housed in specific-pathogen-free conditions. C57B6/J and 129S2/SvPPasCrl mice were obtained from Charles River Laboratories. Nlrp3^tm1Flv^ and Nlrp3^tm1Bhk^ mice have been previously described [20, 27], and were maintained in-house on a C57B6/J background, *Casp1/11*^−^^/−^ mice have been previously described [7], and were maintained in-house on a C57B6/J background. Frozen bone marrow from Nlrp3^tm1Tsch^, which have been previously described [10], was obtained from University of Cologne. *Nlrp3*^fl/fl^ (strain ID: 12809) mice were obtained from Taconic. Rosa26^ERt2Cre^ mice have been previously described [48] and were obtained from Jackson Laboratories (strain ID: 008463). All animal experiments requiring ethical approval were performed under the ethics license AZ. 81-02/04.2019.A336, approved by the Ethics Committee of North Rhein Westphalia. Male and female mice were used, all experiments were sex matched. Genotyping of strain-specific *Nlrp1b* was performed as previously described [6].

### Cell culture

For BMDM production, femurs and tibias were obtained from 8–26-week-old mice and flushed with DMEM + 10% FBS through a 70 μm filter. Isolated cells were either frozen in freezing media (FCS + 10% DMSO), or used fresh. For all individual experiments samples were prepared in a matched manner. Isolated or thawed cells were centrifuged (350 × *g*, 5 min) and resuspended in DMEM + Glutamax, supplemented with 10% FBS, 1% P/S and 15–20% L929 cell-conditioned medium. BMDMs were then differentiated over a period of 7 days (d) in a cell culture incubator (37 °C, 5% CO_2_). Cell culture media was supplemented with an additional 10% of L929 cell-conditioned media on day 3. On the final day of differentiation BMDMs were harvested by cell scraping and resuspended at the desired concentration in DMEM + Glutamax, supplemented with 10% FBS and 1% P/S. For stimulation experiments BMDMs were plated in 96-well plates at a density of 10 × 10^5^/well. For Rosa26^ERt2Cre^ Nlrp3^fl/fl^ BMDMs, Cre was induced on day 4 with 500 nM 4-hydroxytamoxifen. On day 6, media was replaced with fresh complete DMEM.

HEK293 cells stably expressing GFP fused ASC, murine Nlrp1b alleles (a kind gift from Florian I. Schmidt, University Hospital Bonn, DE) and Nlrp1a were cultured in DMEM + Glutamax, supplemented with 10% FBS and 1% P/S. Polyclonal HEK ASC-GFP cell lines expressing NLRP1A-HA under control of the human PGK promoter were generated by lentiviral transduction and selected by blasticidin (10 μg/ml).

### Cell stimulation

BMDMs were plated at a density of 1 × 10^5^ cells/well of a 96-well plate, or 1 × 10^6^ cells/well of a 6-well plate. For RNAseq and LC–MS/MS experiments, BMDMs were cultured in DMEM + Glutamax supplemented with 1% P/S and 10% FCS, and stimulated with LPS-EB Ultrapure (10 ng/ml, Invivogen) for 0, 3 or 24 h. For NLRP1 and NLRP3 inflammasome activation, BMDMs were cultured in DMEM + Glutamax supplemented with 1% P/S and 2% FCS. NLRP1 inflammasome activation by Talabostat (Hoelzel) was induced in BMDMs by the simultaneous treatment with LPS (10 ng/ml) and Talabostat (0.3, 3 or 30 μM) or a DMSO control, for 16 or 24 h, either in presence or absence of VX-765 (50 μM), all as indicated. For long-term inhibition of MCC950 treated BMDMs, cells were cultured in DMEM + Glutamax supplemented with 1% P/S and 10% FCS, supplemented every 48 h with MCC950 (5 μM, Invivogen). NLRP3 inflammasome activation by Nigericin in BMDMs required a priming step where BMDMs were incubated with LPS (10 ng/ml) for 3 h. Subsequently, BMDMs were further incubated with Nigericin (8 μM, Invivogen) for an additional 90 min.

HEK cells were plated at a density of 5 × 10^5^/well of a 12-well plate. NLRP1 inflammasome formation was activated by incubation with Talabostat (0.3, 3 or 30 μM) or a DMSO control for 16 h in DMEM + Glutamax, supplemented with 10% FBS and 1% P/S.

### Immunoblot

Whole cell extracts were lysed in 1X NuPage LDS Sample Buffer (Thermo Fisher), and protein was loaded in each lane of a 4–12% Bolt Bis-Tris Plus Gel (Thermo Fisher), and was then electrophoretically separated, immunoblotted, and imaged with a Sapphire Biomolecular Imager (Azure Biosystems). Primary antibodies used were NLRP3 (Cryo-2, AdipoGen Life Sciences), HA-HRP (2999S, Cell Signaling Technology) and ACTINB-HRP (AB49900, Abcam). Secondary antibodies IRDye 800CW and IRDye 680RD (LI-COR) were used.

### Mouse IL-1β measurements by HTRF

IL-1β concentrations in cell supernatants were measured by a homogenous time-resolved fluorescence (HTRF) “sandwich” antibody-based assay, following manufacturer’s instructions (62MIL1BPEG, CisBio). Briefly, the anti-mouse IL-1β solutions were mixed at a 1:1 ratio. A portion of 4 μl/well of this mixture was distributed in white low-volume medium-binding HTRF-adapted 384-well assay plates (784075, Greiner Bio-One). This was followed by the addition of the samples (tissue culture supernatants; 16 μl/well). The plates were centrifuged at RT, 1000 × *g* for 5 min, followed by a 3 h incubation at RT. HTRF signals were measured using SpectraMax i3.

### LDH cytotoxicity assay

LDH in cell supernatants was measured using the LDH Cytotoxicity Kit (Thermo Fisher) according to manufacturer’s instructions. LDH values were normalised to a total lysis control after substraction of spontaneous background signal. Samples were measured on a SpectraMax i3.

### Peritoneal cavity cell isolation for FACS analysis

Mice were sacrificed by cervical dislocation, and the peritoneal cavity filled with 10 ml phosphate-buffered saline (PBS) + 2 mM EDTA. Mice were subsequently shaken to dislodge residing cells, before the PBS + 2 mM EDTA was removed. Cells were then centrifuged (350 × *g*, 5 min) and resuspended in 1X Red Blood Cell Lysis solution (555899, BD Bioscience) for 15 min at RT. Cells were then centrifuged again (350 × *g*, 5 min) and processed for staining.

### Isolation of bone marrow cells for GMP isolation

In order to isolate GMPs, femurs and tibias were obtained from 8–12-week-old mice and flushed with PBS supplemented with 0.5% BSA and 2 mM EDTA (FACS buffer) through a 70 μm filter. Isolated cells were centrifuged (350 × *g*, 5 min) and resuspended in 1X Red Blood Cell Lysis solution (555899, BD Bioscience) for 15 min at RT. Cells were then centrifuged again (350 × *g*, 5 min) and processed for staining.

### Cell staining for FACS sorting and analysis

All staining was performed at 4 °C in PBS + 1% BSA + 2 mM EDTA. Bone marrow was stained with the following markers for the isolation of GMPs. Lineage markers, all FITC (B220 (11-0452-85, eBioscience) CD19 (11-0193-85, eBioscience), CD11b (11-0112-82, eBioscience), CD3e (11-0033-82, eBioscience), TER-119 (11-5921-85, eBioscience), CD2 (11-0021-85, eBioscience), CD8b (11-0083-85, eBioscience), CD4 (11-0042-85, eBioscience), Ly-6G (553127, BD Pharmingen)), Sca1-Pacific Blue (108120, BioLegend), c-Kit-APC/Cy7 (47-1172-82, eBioscience), CD16/32-PerCP/Cy5.5 (560540, BD Pharmingen) and CD34-AF647 (128606, BioLegend).

For identification of peritoneal cavity cell subsets the following markers were used: CD45-PE/Cy7 (552848, BD Biosciences), CD11b-BV510 (101245, BioLegend), F4/80-APC (123116, BioLegend) and TIM4-PE (130005, BioLegend). Cells were stained in the presence of Fc Block (553142, BD Biosciences).

For ASC speck staining the following markers were used: ASC-PE (653903, BioLegend). Cells were permeabilized with the FoxP3 Transcription Factor Staining Set (00-5523-00, Thermo Fisher) according to manufacturer’s instructions before staining. Cells were stained in the presence of Fc Block (553142, BD Biosciences).

For viability staining in peritoneal cavity populations, cells were incubated with 7-AAD 15 min prior to FACS analysis or sorting. For viability staining in BMDMs LIVE/DEAD Fixable Aqua Dead Cell Stain Kit was used (L34957, Thermo Fisher), and cells stained prior to fixation. For cell number analysis, Precision Plus Counting Beads (Biolegend) were used according to manufacturer’s instructions.

FACS analysis was performed on a BD Canto or Sony ID7000 5L, sorting was performed on a BD Aria III.

### Microglia purification

Microglia were purified from adult brain tissue using the Neural Tissue Dissociation Kit (130-092-628, Miltenyi Biotec), Myelin Removal Beads II (130-096-433, Miltenyi Biotec) and CD11b Microglia Microbeads (130-093-636, Miltenyi Biotec) according to manufacturer’s instructions. Briefly, mice were deeply anaesthetised (Ketamine 240 mg/kg and Xylazine 32 mg/kg bodyweight) and the organs were transcardially perfused with 50 ml cold PBS (pH 7.4). The brain was removed and the two hemispheres separated, only one hemisphere was used for downstream sample preparation. Brain tissue was subsequently digested, myelin was removed and microglia isolated by bead-based positive selection.

### RNA extraction and sequencing

For BMDMs and GMPs RNA was extracted using Picopure RNA Isolation Kit (Thermo Fisher) according to manufacturer’s instructions. For microglia, RNA was extracted using RNeasy Mini Kit (Qiagen). Residual DNA was removed using RNAse-Free DNase Set (Qiagen). RNA was assessed for quality and quantity (TapeStation, Agilent).

For GMPs and BMDMs, RNA sequencing libraries were prepared using the NEBNext Ultra RNA Library Prep Kit for Illumina following manufacturer’s instructions (NEB, Ipswich, MA, USA). Briefly, mRNAs were first enriched with Oligo(dT) beads. Enriched mRNAs were fragmented for 15 min at 94 °C. First-strand and second-strand cDNAs were subsequently synthesised. cDNA fragments were end repaired and adenylated at 3′ ends, and universal adapters were ligated to cDNA fragments, followed by index addition and library enrichment by limited-cycle PCR. Sequencing libraries were validated using NGS Kit on the Agilent 5300 Fragment Analyzer (Agilent Technologies, Palo Alto, CA, USA), and quantified by using Qubit 4.0 Fluorometer (Invitrogen, Carlsbad, CA).

The sequencing libraries were multiplexed and loaded on the flowcell on the Illumina NovaSeq 6000 instrument according to manufacturer’s instructions. The samples were sequenced using a 2 × 150 Pair-End (PE) configuration v1.5. Image analysis and base calling were conducted by the NovaSeq Control Software v1.7 on the NovaSeq instrument. Raw sequence data (.bcl files) generated from Illumina NovaSeq were converted into fastq files and de-multiplexed using Illumina bcl2fastq program version 2.20. One mismatch was allowed for index sequence identification.

For microglia, 3′ mRNA Seq was performed using the QuantSeq 3′ mRNA-Seq Library Prep Kit FWD (Lexogen). Final libraries were pooled and sequenced on an Illumina NovaSeq 6000 device with 1× 100 bp and 10M reads/sample.

### RNAseq analysis

RNAseq datasets were processed with nf-core RNA-seq v3.6 [49] pipeline using STAR [50] for alignment and salmon for gene quantification [51]. The library strandedness parameter was set to forward and the reference was set to GRCm38. Statistical analysis was performed in the R environment [52] with the Bioconductor R-package DESeq2 [53, 54]. The Benjamini–Hochberg method was used to calculate multiple testing adjusted *p* values. For Microglia dataset, only genes with at least three read counts in at least two samples and at least five read counts in total across all samples were considered for analysis. For GMP dataset, only genes with at least 20 read counts in at least three samples and at least 60 read counts in total across all samples were considered for analysis. For BMDM dataset, only genes with at least 50 read counts (minCount) in at least three samples and at least 150 read counts in total across all samples were considered for analysis. Data visualisation, such as volcano plots and heatmaps, were generated upon VST transferred data [55], using R-packages ggplot2 [56], ComplexHeatmap [57] and on Graphpad Prism (v10.0.2).

For the combined genomes approach, the nf-core RNA-seq pipeline (version 3.14.0 [49]) was utilised for the preprocessing and quantification of the RNAseq reads. The pipeline was executed using its default parameters, performing a series of automated steps for quality control, read trimming, alignment and quantification. In our analysis, the mouse reference genome (GRCm38) and the 129S1 genome (129S1_SvImJ_v1) were merged to form a composite reference genome. The trimmed reads were aligned competitively to this composite genome. Competitive alignment ensures that each read is mapped to the genome with the highest sequence similarity, thereby improving the accuracy of the quantification. The resulting count matrix including characteristic identifiers from both genomes, was imported into R (version 4.3 cite R) for downstream analysis. In this step, the counts for each pair of identifiers corresponding to a given gene symbol were aggregated, yielding a single count value per gene symbol. Hence, the gene expression was represented regardless of its source genome.

The differential gene expression analysis was executed with the Bioconductor package DESeq2 (cite DESeq2 and Bioconductor). Subsequently, the Benjamini–Hochberg method was applied to calculate adjusted *p* values (false discovery rate) for each statistical contrast.

Mutation calling was done on RNAseq data with nf-core RnaVar v1.0.0 [49] pipeline using STAR [50] for alignment and GATK4 [58] for variant calling. The group-specific mutations were then identified using isec command from bcftools utilities [59]. Unknown group-specific mutations were removed by overlaying the group-specific mutations with known 129S mutations (Accession Nr: GCA_001624185.1). Gene ontology analysis was performed using gProfiler. Statistical domain scope was defined as all genes or proteins expressed in the given dataset. Significance was determined by Bonferroni correction.

### Sample preparation for mass spectrometry

For proteomics analysis without Anl-enrichment, cells were lysed in sodium deoxycholate (SDC) buffer (1% SDC, 10 mM tris(2-carboxy(ethyl)phosphine), 40 mM 2-chloroacetamide, 100 mM Tris-HCl pH 8.5) heated at 95 °C for 10 min and sonicated to shear DNA. Proteins were digested with trypsin and LysC (1:100 enzyme/protein ratio, w/w) at 37 °C, 1000 rpm overnight. Digests were desalted using in-house-made SDB-RPS StageTips.

Desalted peptides were dried in a vacuum concentrator and resolubilized in 0.1% formic acid. Concentrations were determined using a NanoDrop spectrophotometer and normalised between samples for equal peptide injection.

### LC–MS/MS

LC–MS/MS measurements were performed as previously described [60]. Briefly, peptide mixtures were analysed with an EASY-nLC 1000 or Vanquish chromatographic system coupled to a Orbitrap Exploris 480 (Thermo Fisher Scientific). For Nlrp3^tm1Flv^ litter-mate measurements, 300 ng of peptides were separated on 50 cm in-house-made 75 µm inner diameter columns, packed with 1.9-µm ReproSil C18 beads (Dr. Maisch GmbH) at a flow rate of 300 nl min^−1^ and 60 °C maintained by an in-house-made column oven. Samples were analysed without prefractionation in a single shot measurement with a nonlinear 90 gradient. Spectra were acquired with data-independent acquisition (DIA) using full scans with a range of 300–1650 m/z. For 129S2, C57B6J, C57B6N, Nlrp3^tm1Tsch^, Nlrp3^tm1Bhk^ and Nlrp3^tm1Flv^ measurements, peptides were separated by 120-min chromatographic gradients using a binary buffer system with buffer A (0.1% formic acid in LC–MS-grade water) and buffer B (80% ACN, 0.1% formic acid in LC–MS-grade water), with an Ionopticks Aurora Ultimate analytical column. Samples were measured in DIA mode with a window m/z range from 400 to 1000 m/z separated into 25 isolation windows with a size of 24 m/z per window. We used a staggered window approach with isolation windows shifted by 12 m/z. Data acquisition was controlled by Xcalibur (version 4.4.16.14, Thermo Fisher Scientific).

### LC–MS/MS analysis

For staggered windows, files were deconvoluted with the MSConverter tool of the ProteoWizard software suite (v3.0.21321 [61]). DIA MS raw files were processed by DIA-NN [62] (version 1.8) with FASTA digest for library-free search and deep learning-based spectra, RTs, and IMs prediction enabled. Precursor FDR was set to 1%, and default parameters were used with the following changes: the precursor range was restricted to 300–1650 m/z, or 400–1000 m/z where appropriate, and the fragment ion range to 200–1650 m/z. The “--relaxed-prot-inf” option was enabled via the command line. MBR was enabled, neural network classifier was set to “double-pass mode,” and the quantification strategy to “robust LC (high accuracy).” Spectra were matched against the mouse December 2022 UniProt FASTA database, including protein NL1B1. Protein intensities were normalised by the MaxLFQ [63] algorithm using an in-house script. Bioinformatic analyses were performed with Perseus [64] (version 1.6.15.0) and R (version 4.1.2). Before statistical analysis, quantified proteins were filtered for at least four valid values in at least one group of replicates. Samples were identified as outliers by principal component analysis and subsequently removed if necessary. Statistical tests and parameters used to evaluate annotation enrichment and significant abundance differences of quantified proteins are specified in the figure legends.

### Statistical analysis

Statistical analyses and *n* numbers are provided in the appropriate figure legends. All experiments were performed with a minimum of three biological replicates. Experiments were performed a minimum of two independent times, with the exception of Figs. 1E, F, 5E and 6B, E. Data are presented as mean ± standard deviation. A difference was considered statistically significant at *q* < 0.05. Statistical analyses were performed by Graphpad Prism (v10.2). Statistical analysis for RNAseq and LC–MS/MS is outlined in the relevant “Materials and methods” section.

## Supplementary information

Supplemental Figures and raw westerns

Supplemental Table 1. Nlrp3^tm1Flv^ BMDM RNAseq

Supplemental Table 2. Nlrp3^tm1Flv^ GMP RNAseq

Supplemental Table 3. Nlrpt3^tm1Flv^ (Nlrp3KO) BMDM 129S SNPs

Supplemental Table 4. Nlrpt3^tm1Flv^ (Nlrp3WT) BMDM 129S SNPs

Supplemental Table 5. Nlrpt3^tm1Flv^ (Nlrp3KO) GMP 129S SNPs

Supplemental Table 6. Nlrp3^tm1Flv^ Microglia RNAseq

Supplemental Table 7. Nlrp3^tm1Flv^ BMDM Combined Genomes RNAseq

Supplemental Table 8. Nlrp3^tm1Flv^ BMDM LC-MS/MS

Supplemental Table 9. Nlrp3 strains LC-MS/MS

Supplemental Table 10. 129S C57B6 LC-MS/MS

## Data Availability

Raw RNAseq data generated in this study have been deposited to the Array Express and are available under the accession number E-MTAB-13601. Raw LC–MS/MS data have been deposited to the ProteomeXchange Consortium via the PRIDE partner repository, with the dataset identifier PXD046782.

## References

[1] Lilue J, Doran AG, Fiddes IT, Abrudan M, Armstrong J, Bennett R, et al. Sixteen diverse laboratory mouse reference genomes define strain-specific haplotypes and novel functional loci. Nat Genet. 2018;50:1574–83. 30275530 10.1038/s41588-018-0223-8 PMC6205630

[2] Li P, Allen H, Banerjee S, Franklin S, Herzog L, Johnston C, et al. Mice deficient in IL-1β-converting enzyme are defective in production of mature IL-1β and resistant to endotoxic shock. Cell. 1995;80:401–11. 7859282 10.1016/0092-8674(95)90490-5

[3] Chisolm DA, Cheng W, Colburn SA, Silva-Sanchez A, Meza-Perez S, Randall TD, et al. Defining genetic variation in widely used congenic and backcrossed mouse models reveals varied regulation of genes important for immune responses. Immunity. 2019;51:155–168.e5. 31248780 10.1016/j.immuni.2019.05.006 PMC6883924

[4] Vanden Berghe T, Hulpiau P, Martens L, Vandenbroucke RE, Van Wonterghem E, Perry SW, et al. Passenger mutations confound interpretation of all genetically modified congenic mice. Immunity. 2015;43:200–9. 26163370 10.1016/j.immuni.2015.06.011 PMC4800811

[5] Er-Lukowiak M, Duan Y, Rassendren F, Ulmann L, Nicke A, Ufer F, et al. A P2rx7 passenger mutation affects the vitality and function of T cells in congenic mice. iScience. 2020;23:101870. 33336163 10.1016/j.isci.2020.101870 PMC7733020

[6] Gerlic M, Croker BA, Cengia LH, Moayeri M, Kile BT, Masters SL. NLRP1a expression in Srebp-1a-deficient mice. Cell Metab. 2014;19:345–6. 24606891 10.1016/j.cmet.2014.02.002

[7] Kayagaki N, Warming S, Lamkanfi M, Walle LV, Louie S, Dong J, et al. Non-canonical inflammasome activation targets caspase-11. Nature. 2011;479:117–21. 22002608 10.1038/nature10558

[8] Taabazuing CY, Griswold AR, Bachovchin DA. The NLRP1 and CARD8 inflammasomes. Immunol Rev. 2020;297:13–25. 32558991 10.1111/imr.12884 PMC7483925

[9] Boyden ED, Dietrich WF. Nalp1b controls mouse macrophage susceptibility to anthrax lethal toxin. Nat Genet. 2006;38:240–4. 16429160 10.1038/ng1724

[10] Martinon F, Pétrilli V, Mayor A, Tardivel A, Tschopp J. Gout-associated uric acid crystals activate the NALP3 inflammasome. Nature. 2006;440:237–41. 16407889 10.1038/nature04516

[11] Duewell P, Kono H, Rayner KJ, Sirois CM, Vladimer G, Bauernfeind FG, et al. NLRP3 inflammasomes are required for atherogenesis and activated by cholesterol crystals. Nature. 2010;464:1357–61. 20428172 10.1038/nature08938 PMC2946640

[12] Coll RC, Robertson AAB, Chae JJ, Higgins SC, Muñoz-Planillo R, Inserra MC, et al. A small-molecule inhibitor of the NLRP3 inflammasome for the treatment of inflammatory diseases. Nat Med. 2015;21:248–55. 25686105 10.1038/nm.3806 PMC4392179

[13] Inoue M, Chen P, Siecinski S, Li Q, Liu C, Steinman L, et al. An interferon-β-resistant and NLRP3 inflammasome–independent subtype of EAE with neuronal damage. Nat Neurosci. 2016;19:1599–609. 27820602 10.1038/nn.4421 PMC5482232

[14] Ising C, Venegas C, Zhang S, Scheiblich H, Schmidt SV, Vieira-Saecker A, et al. NLRP3 inflammasome activation drives tau pathology. Nature. 2019;575:669–73. 31748742 10.1038/s41586-019-1769-z PMC7324015

[15] Heneka MT, Kummer MP, Stutz A, Delekate A, Schwartz S, Vieira-Saecker A, et al. NLRP3 is activated in Alzheimer’s disease and contributes to pathology in APP/PS1 mice. Nature. 2013;493:674–8. 23254930 10.1038/nature11729 PMC3812809

[16] Christ A, Günther P, Lauterbach MAR, Duewell P, Biswas D, Pelka K, et al. Western diet triggers NLRP3-dependent innate immune reprogramming. Cell. 2018;172:162–175.e14. 29328911 10.1016/j.cell.2017.12.013 PMC6324559

[17] van Deventer HW, Burgents JE, Wu QP, Woodford R-MT, Brickey WJ, Allen IC, et al. The inflammasome component Nlrp3 impairs antitumor vaccine by enhancing the accumulation of tumor-associated myeloid-derived suppressor cells. Cancer Res. 2010;70:10161–9. 21159638 10.1158/0008-5472.CAN-10-1921 PMC3059219

[18] Gris D, Ye Z, Iocca HA, Wen H, Craven RR, Gris P, et al. NLRP3 plays a critical role in the development of experimental autoimmune encephalomyelitis by mediating Th1 and Th17 responses. J Immunol. 2010;185:974–81. 20574004 10.4049/jimmunol.0904145 PMC3593010

[19] Irrera N, Pizzino G, Calò M, Pallio G, Mannino F, Famà F, et al. Lack of the Nlrp3 inflammasome improves mice recovery following traumatic brain injury. Front Pharmacol. 2017;8:459. 28769794 10.3389/fphar.2017.00459 PMC5509758

[20] Sutterwala FS, Ogura Y, Szczepanik M, Lara-Tejero M, Lichtenberger GS, Grant EP, et al. Critical role for NALP3/CIAS1/Cryopyrin in innate and adaptive immunity through its regulation of caspase-1. Immunity. 2006;24:317–27. 16546100 10.1016/j.immuni.2006.02.004

[21] Chui AJ, Okondo MC, Rao SD, Gai K, Griswold AR, Johnson DC, et al. N-terminal degradation activates the NLRP1B inflammasome. Science. 2019;364:82–85. 30872531 10.1126/science.aau1208 PMC6610862

[22] Zhong FL, Robinson K, Teo DET, Tan K-Y, Lim C, Harapas CR, et al. Human DPP9 represses NLRP1 inflammasome and protects against autoinflammatory diseases via both peptidase activity and FIIND domain binding. J Biol Chem. 2018;293:18864–78. 30291141 10.1074/jbc.RA118.004350 PMC6295727

[23] Vasconcelos, de NM, Vliegen G, Gonçalves A, Hert ED, Martín-Pérez R, et al. DPP8/DPP9 inhibition elicits canonical Nlrp1b inflammasome hallmarks in murine macrophages. Life Sci Alliance. 2019;2:e201900313. 30718379 10.26508/lsa.201900313 PMC6362307

[24] Okondo MC, Rao SD, Taabazuing CY, Chui AJ, Poplawski SE, Johnson DC, et al. Inhibition of Dpp8/9 activates the Nlrp1b inflammasome. Cell Chem Biol. 2018;25:262–267.e5. 29396289 10.1016/j.chembiol.2017.12.013 PMC5856610

[25] Gai K, Okondo MC, Rao SD, Chui AJ, Ball DP, Johnson DC, et al. DPP8/9 inhibitors are universal activators of functional NLRP1 alleles. Cell Death Dis. 2019;10:1–10. 10.1038/s41419-019-1817-5 PMC6683174 31383852

[26] van der Veeken J, Zhong Y, Sharma R, Mazutis L, Dao P, Pe’er D, et al. Natural genetic variation reveals key features of epigenetic and transcriptional memory in virus-specific CD8 T cells. Immunity. 2019;50:1202–1217.e7. 31027997 10.1016/j.immuni.2019.03.031 PMC7023907

[27] Kovarova M, Hesker PR, Jania L, Nguyen M, Snouwaert JN, Xiang Z, et al. NLRP1-dependent pyroptosis leads to acute lung injury and morbidity in mice. J Immunol. 2012;189:2006–16. 22753929 10.4049/jimmunol.1201065 PMC3635067

[28] Jenster L-M, Lange K-E, Normann S, vom Hemdt A, Wuerth JD, Schiffelers LDJ, et al. P38 kinases mediate NLRP1 inflammasome activation after ribotoxic stress response and virus infection. J Exp Med. 2023;220:e20220837. 36315050 10.1084/jem.20220837 PMC9623368

[29] Zhou T-A, Hsu H-P, Tu Y-H, Cheng H-K, Lin C-Y, Chen N-J, et al. Thymic macrophages consist of two populations with distinct localization and origin. eLife. 2022;11:e75148. 36449334 10.7554/eLife.75148 PMC9754631

[30] Thornley TB, Fang Z, Balasubramanian S, Larocca RA, Gong W, Gupta S, et al. Fragile TIM-4–expressing tissue resident macrophages are migratory and immunoregulatory. J Clin Investig. 2014;124:3443–54. 24983317 10.1172/JCI73527 PMC4109530

[31] Silva HM, Báfica A, Rodrigues-Luiz GF, Chi J, Santos P, d’Emery A, et al. Vasculature-associated fat macrophages readily adapt to inflammatory and metabolic challenges. J Exp Med. 2019;216:786–806. 30862706 10.1084/jem.20181049 PMC6446877

[32] Tran S, Baba I, Poupel L, Dussaud S, Moreau M, Gélineau A, et al. Impaired Kupffer cell self-renewal alters the liver response to lipid overload during non-alcoholic steatohepatitis. Immunity. 2020;53:627–640.e5. 32562600 10.1016/j.immuni.2020.06.003

[33] Shaw TN, Houston SA, Wemyss K, Bridgeman HM, Barbera TA, Zangerle-Murray T, et al. Tissue-resident macrophages in the intestine are long lived and defined by Tim-4 and CD4 expression. J Exp Med. 2018;215:1507–18. 29789388 10.1084/jem.20180019 PMC5987925

[34] Louwe PA, Badiola Gomez L, Webster H, Perona-Wright G, Bain CC, Forbes SJ, et al. Recruited macrophages that colonize the post-inflammatory peritoneal niche convert into functionally divergent resident cells. Nat Commun. 2021;12:1770. 33741914 10.1038/s41467-021-21778-0 PMC7979918

[35] Ewald SE, Chavarria-Smith J, Boothroyd JC. NLRP1 is an inflammasome sensor for *Toxoplasma gondii*. Infect Immun. 2014;82:460–8. 24218483 10.1128/IAI.01170-13 PMC3911858

[36] Kobayashi N, Karisola P, Peña-Cruz V, Dorfman DM, Jinushi M, Umetsu SE, et al. TIM-1 and TIM-4 glycoproteins bind phosphatidylserine and mediate uptake of apoptotic cells. Immunity. 2007;27:927–40. 18082433 10.1016/j.immuni.2007.11.011 PMC2757006

[37] Czuczman MA, Fattouh R, van Rijn JM, Canadien V, Osborne S, Muise AM, et al. Listeria monocytogenes exploits efferocytosis to promote cell-to-cell spread. Nature. 2014;509:230–4. 24739967 10.1038/nature13168 PMC4151619

[38] Min C, Park J, Kim G, Moon H, Lee S-A, Kim D, et al. Tim-4 functions as a scavenger receptor for phagocytosis of exogenous particles. Cell Death Dis. 2020;11:1–10. 32703939 10.1038/s41419-020-02773-7 PMC7378189

[39] Wang Y, Wang Y, Ding L, Ren X, Wang B, Wang L, et al. Tim-4 reprograms cholesterol metabolism to suppress antiviral innate immunity by disturbing the Insig1-SCAP interaction in macrophages. Cell Rep. 2022;41:111738. 36450259 10.1016/j.celrep.2022.111738

[40] Magalhaes MS, Smith P, Portman JR, Jackson-Jones LH, Bain CC, Ramachandran P, et al. Role of Tim4 in the regulation of ABCA1+ adipose tissue macrophages and post-prandial cholesterol levels. Nat Commun. 2021;12:4434. 34290249 10.1038/s41467-021-24684-7 PMC8295389

[41] Joshi S, López L, Morosi LG, Amadio R, Pachauri M, Bestagno M, et al. Tim4 enables large peritoneal macrophages to cross-present tumor antigens at early stages of tumorigenesis. Cell Rep. 2024;43:114096. 38607919 10.1016/j.celrep.2024.114096

[42] Collazo CM, Yap GS, Sempowski GD, Lusby KC, Tessarollo L, Vande Woude GF, et al. Inactivation of LRG-47 and IRG-47 reveals a family of interferon γ-inducible genes with essential, pathogen-specific roles in resistance to infection. J Exp Med. 2001;194:181–7. 11457893 10.1084/jem.194.2.181 PMC2193451

[43] Mukundan L, Odegaard JI, Morel CR, Heredia JE, Mwangi JW, Ricardo-Gonzalez RR, et al. PPAR-Δ senses and orchestrates clearance of apoptotic cells to promote tolerance. Nat Med. 2009;15:1266–72. 19838202 10.1038/nm.2048 PMC2783696

[44] Coombes JL, Siddiqui KRR, Arancibia-Cárcamo CV, Hall J, Sun C-M, Belkaid Y, et al. A functionally specialized population of mucosal CD103+ DCs induces Foxp3+ regulatory T cells via a TGF-β -and retinoic acid-dependent mechanism. J Exp Med. 2007;204:1757–64. 17620361 10.1084/jem.20070590 PMC2118683

[45] MacKay LK, Rahimpour A, Ma JZ, Collins N, Stock AT, Hafon M-L, et al. The developmental pathway for CD103+ CD8+ tissue-resident memory T cells of skin. Nat Immunol. 2013;14:1294–301. 24162776 10.1038/ni.2744

[46] Liston P, Fong WG, Kelly NL, Toji S, Miyazaki T, Conte D, et al. Identification of XAF1 as an antagonist of XIAP anti-caspase activity. Nat Cell Biol. 2001;3:128–33. 11175744 10.1038/35055027

[47] Mariathasan S, Weiss DS, Newton K, McBride J, O’Rourke K, Roose-Girma M, et al. Cryopyrin activates the inflammasome in response to toxins and ATP. Nature. 2006;440:228–32. 16407890 10.1038/nature04515

[48] Ventura A, Kirsch DG, McLaughlin ME, Tuveson DA, Grimm J, Lintault L, et al. Restoration of p53 function leads to tumour regression in vivo. Nature. 2007;445:661–5. 17251932 10.1038/nature05541

[49] Ewels PA, Peltzer A, Fillinger S, Patel H, Alneberg J, Wilm A, et al. The nf-core framework for community-curated bioinformatics pipelines. Nat Biotechnol. 2020;38:276–8. 32055031 10.1038/s41587-020-0439-x

[50] Dobin A, Davis CA, Schlesinger F, Drenkow J, Zaleski C, Jha S, et al. STAR: ultrafast universal RNA-seq aligner. Bioinformatics. 2013;29:15–21. 23104886 10.1093/bioinformatics/bts635 PMC3530905

[51] Patro R, Duggal G, Love MI, Irizarry RA, Kingsford C. Salmon: fast and bias-aware quantification of transcript expression using dual-phase inference. Nat Methods. 2017. 10.1038/nmeth.4197. 10.1038/nmeth.4197 PMC5600148 28263959

[52] R Core Team. R: the R project for statistical computing. 2019. https://www.r-project.org/.

[53] Love MI, Huber W, Anders S. Moderated estimation of fold change and dispersion for RNA-seq data with DESeq2. Genome Biol. 2014. 10.1186/s13059-014-0550-8. 10.1186/s13059-014-0550-8 PMC4302049 25516281

[54] Huber W, Carey VJ, Gentleman R, Anders S, Carlson M, Carvalho BS, et al. Orchestrating high-throughput genomic analysis with Bioconductor. Nat Methods. 2015;12:115–21. 25633503 10.1038/nmeth.3252 PMC4509590

[55] Anders S, Huber W. Differential expression analysis for sequence count data. Genome Biol. 2010;11:1–12. 10.1186/gb-2010-11-10-r106 PMC3218662 20979621

[56] Wickham H. Elegant graphics for data analysis. 2016. https://ggplot2-book.org/.

[57] Gu Z, Eils R, Schlesner M. Complex heatmaps reveal patterns and correlations in multidimensional genomic data. Bioinformatics. 2016;32:2847–9. 27207943 10.1093/bioinformatics/btw313

[58] Heldenbrand JR, Baheti S, Bockol MA, Drucker TM, Hart SN, Hudson ME, et al. Recommendations for performance optimizations when using GATK3.8 and GATK4. BMC Bioinform. 2019. 10.1186/s12859-019-3169-7. 10.1186/s12859-019-3169-7 PMC6842142 31703611

[59] Li H, Barrett J. A statistical framework for SNP calling, mutation discovery, association mapping and population genetical parameter estimation from sequencing data. Bioinformatics. 2011;27:2987–93. 21903627 10.1093/bioinformatics/btr509 PMC3198575

[60] Swietlik JJ, Bärthel S, Falcomatà C, Fink D, Sinha A, Cheng J, et al. Cell-selective proteomics segregates pancreatic cancer subtypes by extracellular proteins in tumors and circulation. Nat Commun. 2023;14:2642. 37156840 10.1038/s41467-023-38171-8 PMC10167354

[61] Chambers MC, Maclean B, Burke R, Amodei D, Ruderman DL, Neumann S, et al. A cross-platform toolkit for mass spectrometry and proteomics. Nat Biotechnol. 2012;30:918–20. 23051804 10.1038/nbt.2377 PMC3471674

[62] Demichev V, Messner, CB, Vernardis SI, Lilley KS, Ralser M. DIA-NN: neural networks and interference correction enable deep proteome coverage in high throughput. Nat Methods. 2020;17:41–4. 10.1038/s41592-019-0638-x PMC6949130 31768060

[63] Cox J, Hein MY, Luber CA, Paron I, Nagaraj N, Mann M. Accurate proteome-wide label-free quantification by delayed normalization and maximal peptide ratio extraction, termed MaxLFQ. Mol Cell Proteom. 2014;13:2513–26. 10.1074/mcp.M113.031591 PMC4159666 24942700

[64] Tyanova S, Temu T, Sinitcyn P, Carlson A, Hein MY, Geiger T, et al. The Perseus computational platform for comprehensive analysis of (prote)omics data. Nat Methods. 2016;13:731–40. 27348712 10.1038/nmeth.3901

