# Supplementary material for: Retention of ES cell-derived 129S genome drives NLRP1 hypersensitivity and transcriptional deregulation in Nlrp3tm1Flv mice
Source: Cell Death Differ. 2024 Sep 17;31(12):1717–29. doi: 10.1038/s41418-024-01379-2 (PMC11618613; doi:10.1038/s41418-024-01379-2)
Supplement: Supplementary file 1 — Supplemental Figures and raw westerns [file 41418_2024_1379_MOESM1_ESM.pdf]

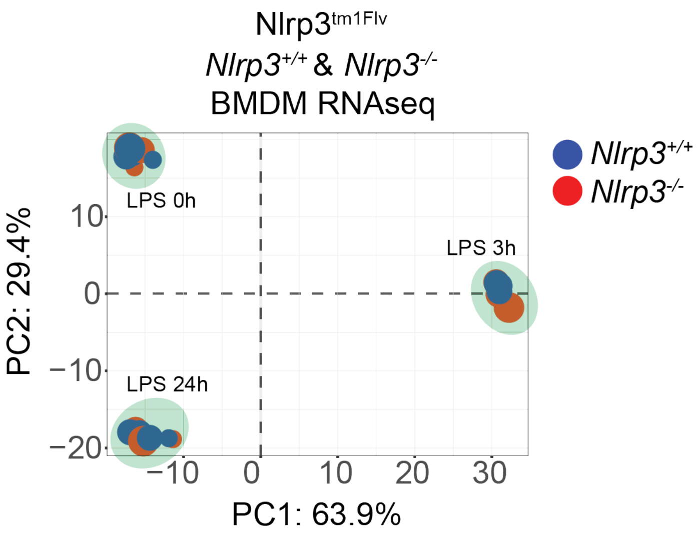


Supplemental Figure 1. **PCA of Nlrp3$^{tm1Flv}$ *Nlrp3$^{+/+}$* and *Nlrp3$^{-/-}$* BMDMs RNAseq**

Principal component analysis of RNAseq analysis of Nlrp3$^{tm1Flv}$ *Nlrp3$^{+/+}$* and *Nlrp3$^{-/-}$* BMDMs at baseline and following LPS stimulation.

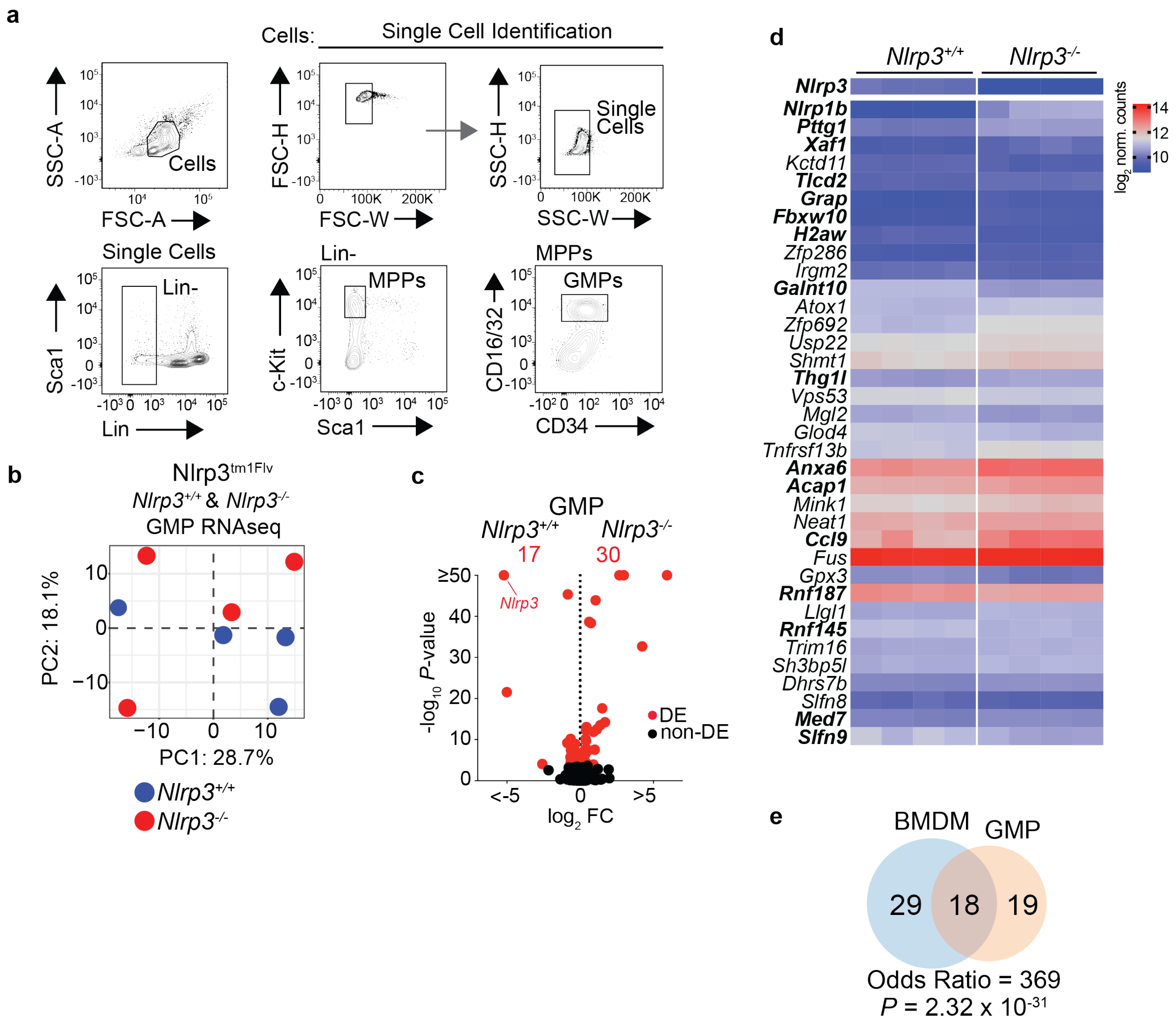


Supplemental Figure 2. **FACS isolation and RNAseq of GMPs from Nlrp3$^{tm1Flv}$ *Nlrp3$^{+/+}$* and *Nlrp3$^{-/-}$* mice**

**a**. Flow cytometry plots representing gating strategy for sorting of GMPs from mouse bone marrow.

**b.** Principal component analysis of RNAseq analysis of *Nlrp3$^{+/+}$* and *Nlrp3$^{-/-}$* GMPs.

**c**. Volcano plot of gene expression fold-change vs. *P*-value in RNA-seq of *Nlrp3$^{+/+}$* and *Nlrp3$^{-/-}$* GMPs. Total number of differentially expressed (DE, adj. $P < 0.05$, Benjamini-Hochberg adjusted) genes, and individual DE genes, are shown in red.

**d**. Heatmap of $\log_2$ normalised counts of significantly differentially expressed (adj. $P <$ 0.05) protein coding genes in *Nlrp3$^{-/-}$* GMPs. Differentially expressed genes shared with *Nlrp3$^{-/-}$*BMDMs are shown in bold.

**e**. Venn diagram showing enrichment of shared DE genes (adj. $P < 0.05$) between *Nlrp3$^{+/+}$* and *Nlrp3$^{-/-}$* in BMDMs and GMPs. One-sided Fisher's exact test was applied for the odds ratio and *P* value.

**a**

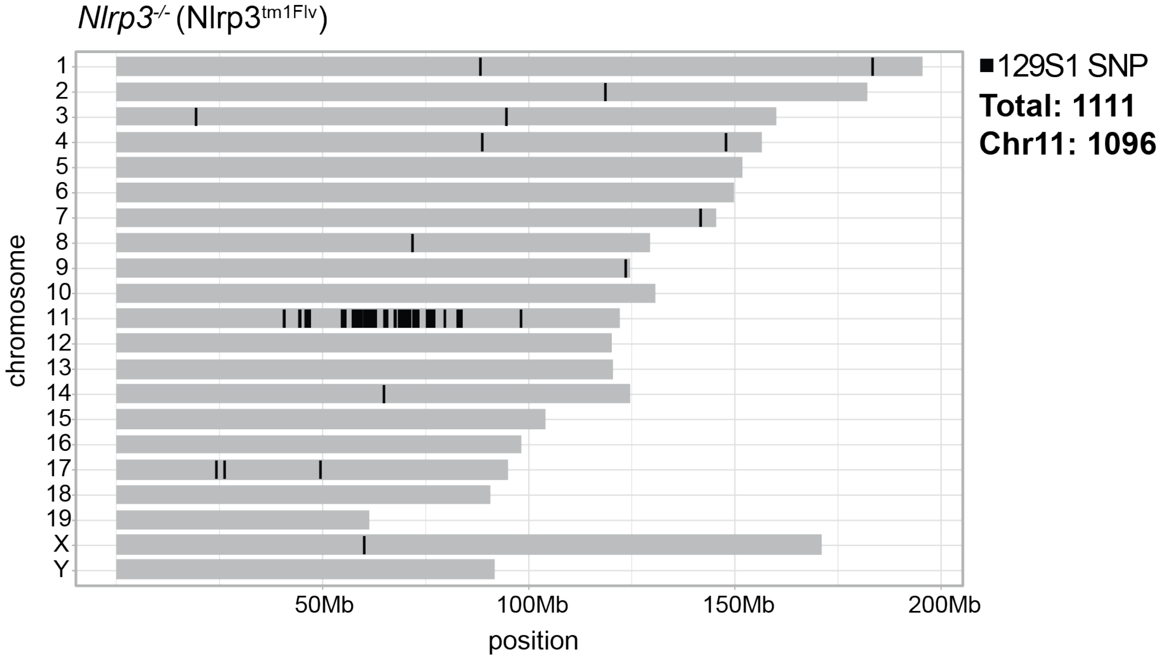


**b**

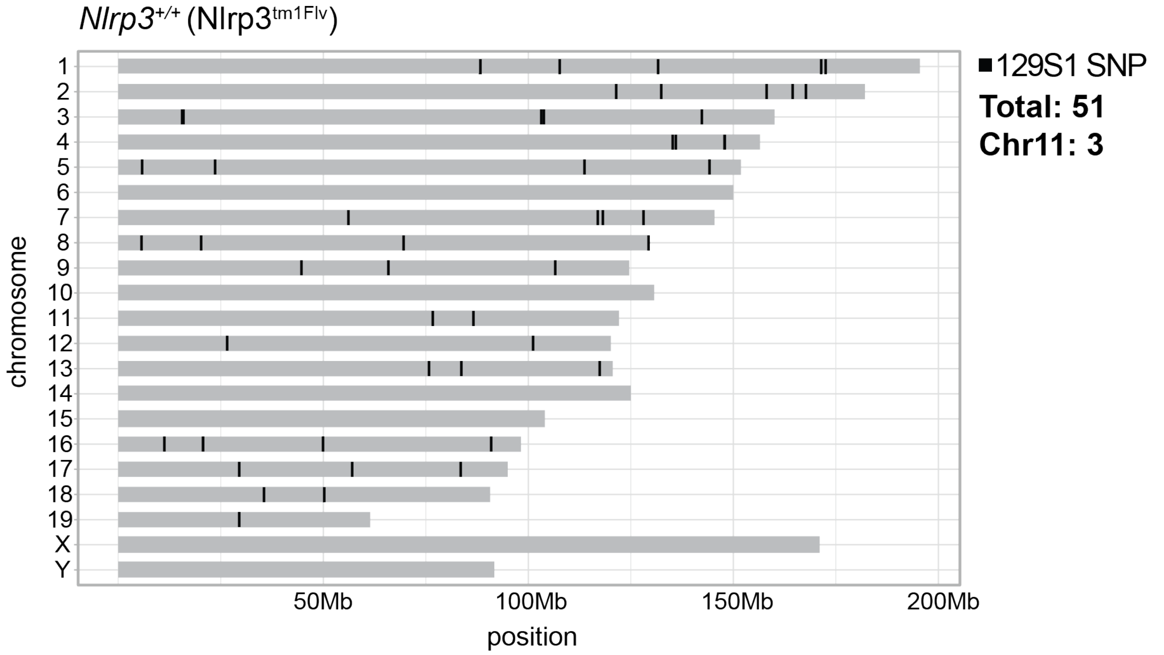


Supplemental Figure 3. **Genomic position of identified 129S1 SNPs in Nlrp3$^{tm1Flv}$ *Nlrp3$^{+/+}$* and *Nlrp3$^{-/-}$* BMDMs**

**a.** Line plot showing every identified SNP mapping to the 129S1 genome compared to the reference genome in RNAseq data from *Nlrp3$^{-/-}$* BMDMs.

**b**. Line plot showing every identified SNP mapping to the 129S1 genome compared to the reference genome in RNAseq data from *Nlrp3$^{+/+}$* BMDMs.

**a-b**. Presence of SNPs based on identification in at least 3 out of 4 biological replicates. Total number of identified SNPs shown on righthand side.

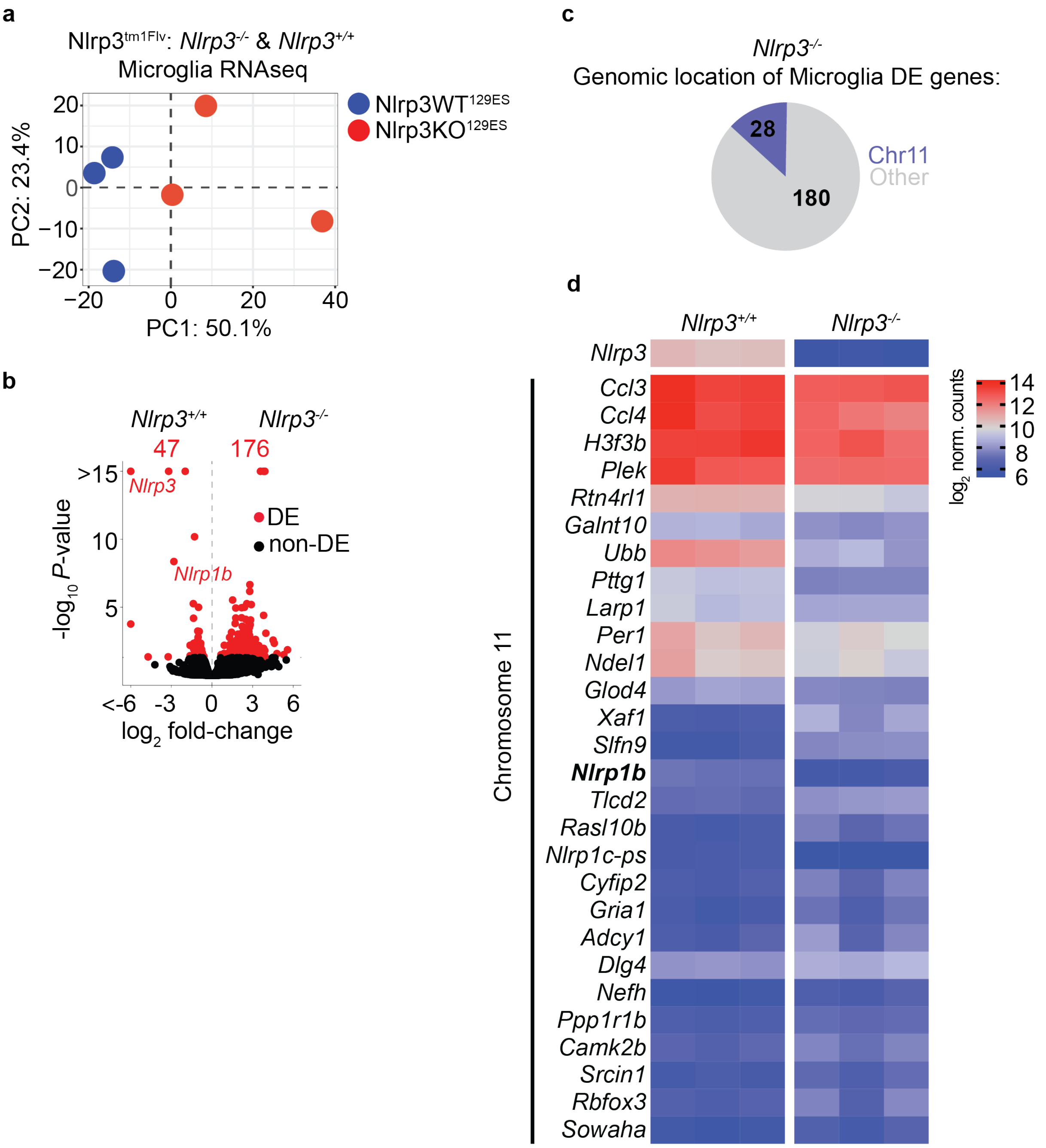


Supplemental Figure 4. **Gene expression analysis of Nlrp3$^{tm1Flv}$ *Nlrp3$^{-/-}$* and *Nlrp3$^{+/+}$* microglia**

**a.** Principal component analysis of RNAseq analysis of *Nlrp3$^{+/+}$* and *Nlrp3$^{-/-}$* microglia.

**b**. Volcano plot of gene expression fold-change vs. *P*-value in RNA-seq of *Nlrp3$^{+/+}$* and *Nlrp3$^{-/-}$* microglia. Total number of differentially expressed (DE, adj. $P < 0.05$, Wald Test, Benjamini-Hochberg adjusted) genes, and individual DE genes, are shown in red.

**c**. Pie chart showing the number of differentially expressed (DE) genes (adj. $P < 0.05$) in microglia between *Nlrp3$^{+/+}$* and *Nlrp3$^{-/-}$* mice, and whether they are located on chromosome 11 (blue) or an alternative chromosome (grey).

**d**. Heatmap of $log_2$ normalised counts of significantly differentially expressed genes (adj. $P < 0.05$) located on Chromosome 11 between *Nlrp3*$^{+/+}$ and *Nlrp3*$^{-/-}$ microglia.

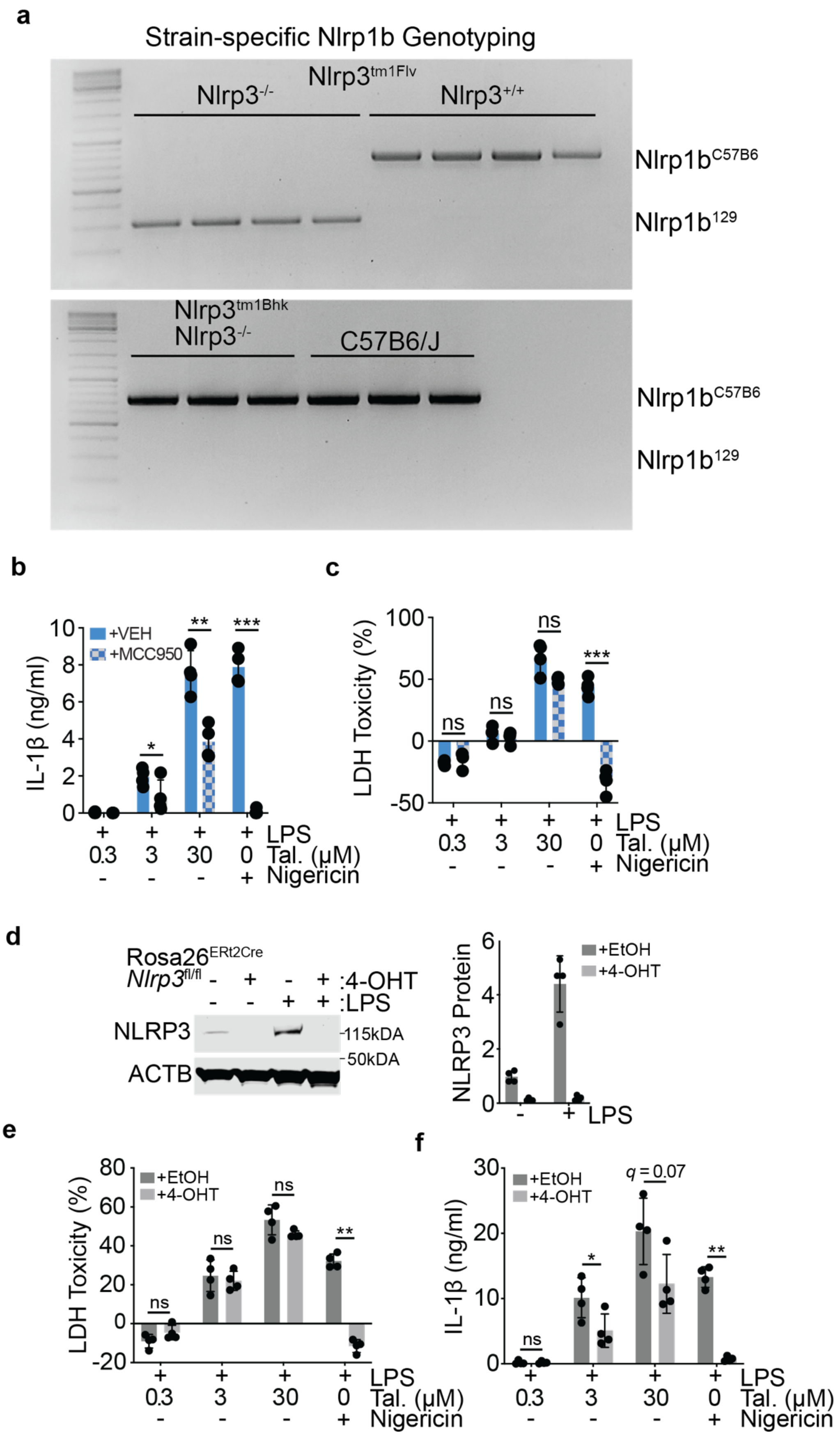

a
Strain-specific Nlrp1b Genotyping
Nlrp3tm1Flv
Nlrp3-/-
Nlrp3+/+
Nlrp1bC57B6
Nlrp1b129
Nlrp3tm1Bhk
Nlrp3-/-
C57B6/J
Nlrp1bC57B6
Nlrp1b129
b
IL-1β (ng/ml)
+VEH
+MCC950
LPS
Tal. (μM)
Nigericin
c
LDH Toxicity (%)
LPS
Tal. (μM)
Nigericin
d
Rosa26ERt2Cre
Nlrp3fl/fl
:4-OHT
:LPS
NLRP3
ACTB
115kDA
50kDA
NLRP3 Protein
+EtOH
+4-OHT
LPS
e
LDH Toxicity (%)
+EtOH
+4-OHT
LPS
Tal. (μM)
Nigericin
f
IL-1β (ng/ml)
+EtOH
+4-OHT
q = 0.07
LPS
Tal. (μM)
Nigericin

Supplemental Figure 5. **Nlrp3$^{tm1Bhk}$ mice contain Nlrp1$^{C57B6}$ locus, while pharmacological inhibition, and inducible NLRP3 degradation show a role for NLRP3 in Talabostat induced IL-1β release**

**a.** Genotyping analysis of Nlrp1b strain from Nlrp3$^{tm1Flv}$, Nlrp3$^{tm1Bhk}$ and C57B6/J mice

**b.** IL-1β release from C57B6/J BMDMs +/-MCC950 after stimulation with LPS (10ng/ml) and Talabostat (0.3 μM, 3 μM or 30 μM) for 24 hours.

**c.** LDH release, relative to total lysis controls, from C57B6/J BMDMs +/-MCC950 after stimulation with LPS (10ng/ml) and Talabostat (0.3 μM, 3 μM or 30 μM) for 24 hours.

**d.** Analysis of NLRP3 expression in Rosa26$^{ERt2Cre}$ *Nlrp3*$^{fl/fl}$ BMDMs treated with EtOH or 4-OHT, +/- LPS. Left: Western blot of NLRP3 and ACTB. Right: quantification of NLRP3 protein expression relative to ACTB.

**e.** Analysis of LDH release, relative to total lysis controls, from Rosa26$^{ERt2Cre}$ *Nlrp3*$^{fl/fl}$ BMDMs treated with EtOH or 4-OHT, and co-stimulated with LPS (10ng/ml) and Talabostat (0.3 μM, 3 μM or 30 μM), or LPS (10ng/ml, 3 hours) + Nigericin (8 μM, 90 minutes).

**f.** Analysis of IL-1β release from Rosa26$^{ERt2Cre}$ *Nlrp3*$^{fl/fl}$ BMDMs treated with EtOH or 4-OHT, and co-stimulated with LPS (10ng/ml) and Talabostat (0.3 μM, 3 μM or 30 μM), or LPS (10ng/ml, 3 hours) + Nigericin (8 μM, 90 minutes).

**b – e.** *P* values were calculated using multiple unpaired parametric t-tests. FDR ($q$) was calculated using Benjamini Hochberg correction. * = $q < 0.05$, ** = $q < 0.01$, *** = $q < 0.001$, **** = $q < 0.0001$, ns = not significant ($q > 0.05$). Error bars represent standard deviation.

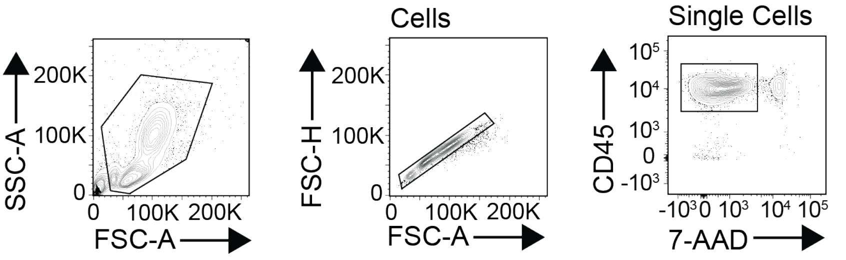


Supplemental Figure 6. **FACS identification of live CD45+ cells in the peritoneum**

Flow cytometry plots representing gating strategy for identification of CD45+ Live cells from peritoneal cavity

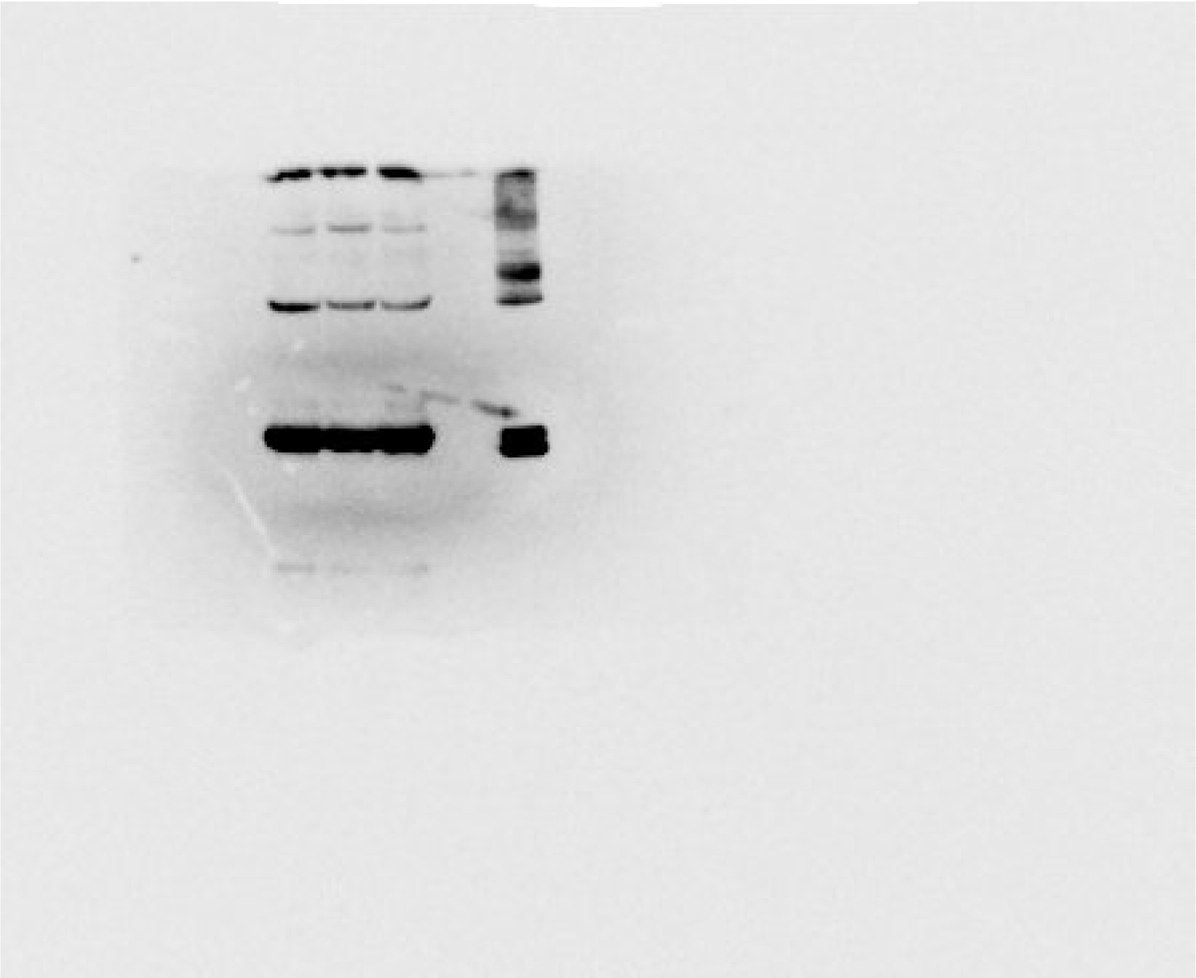

Raw western blot of ACTB staining, pertaining to Figure 5G

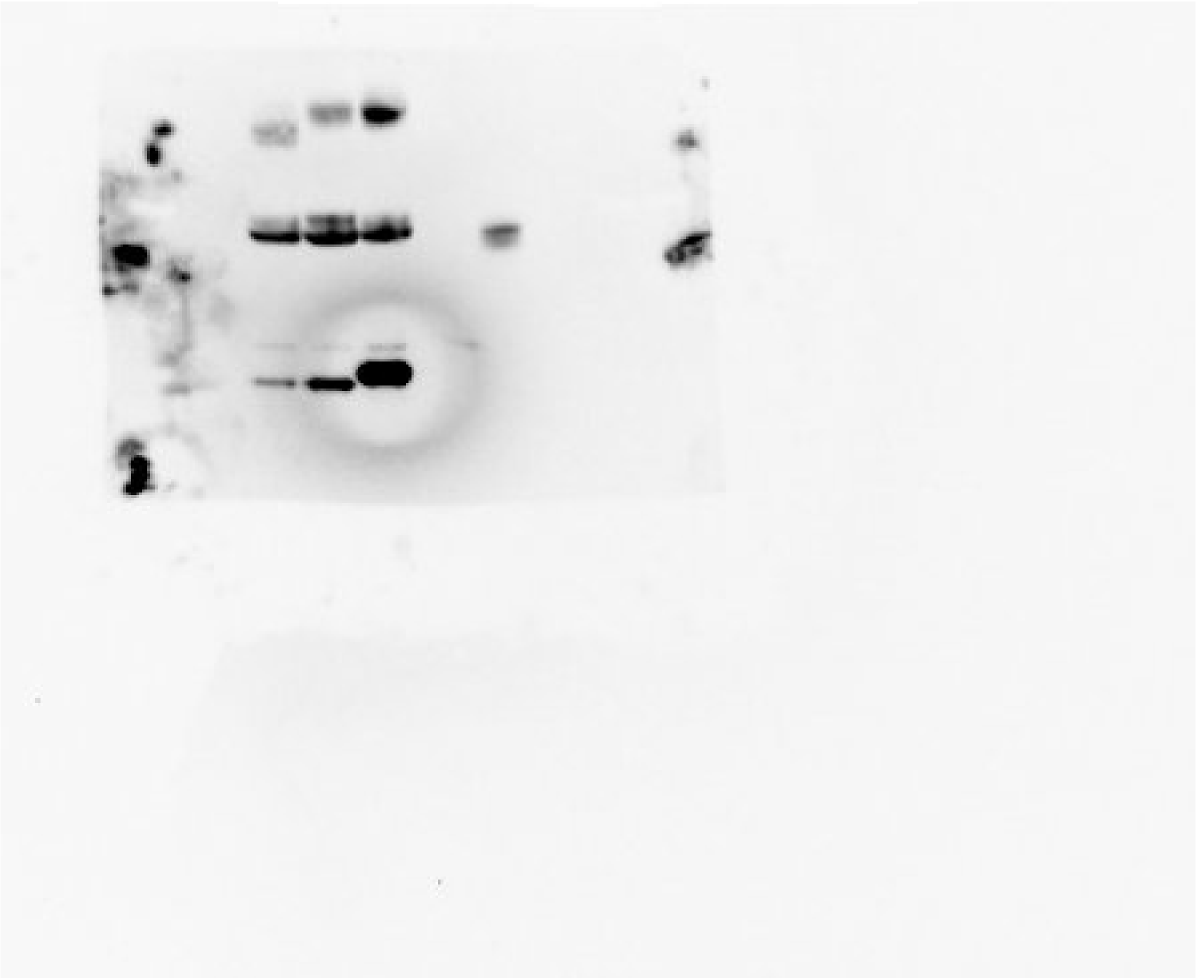

Raw western blot of HA staining, pertaining to Figure 5G

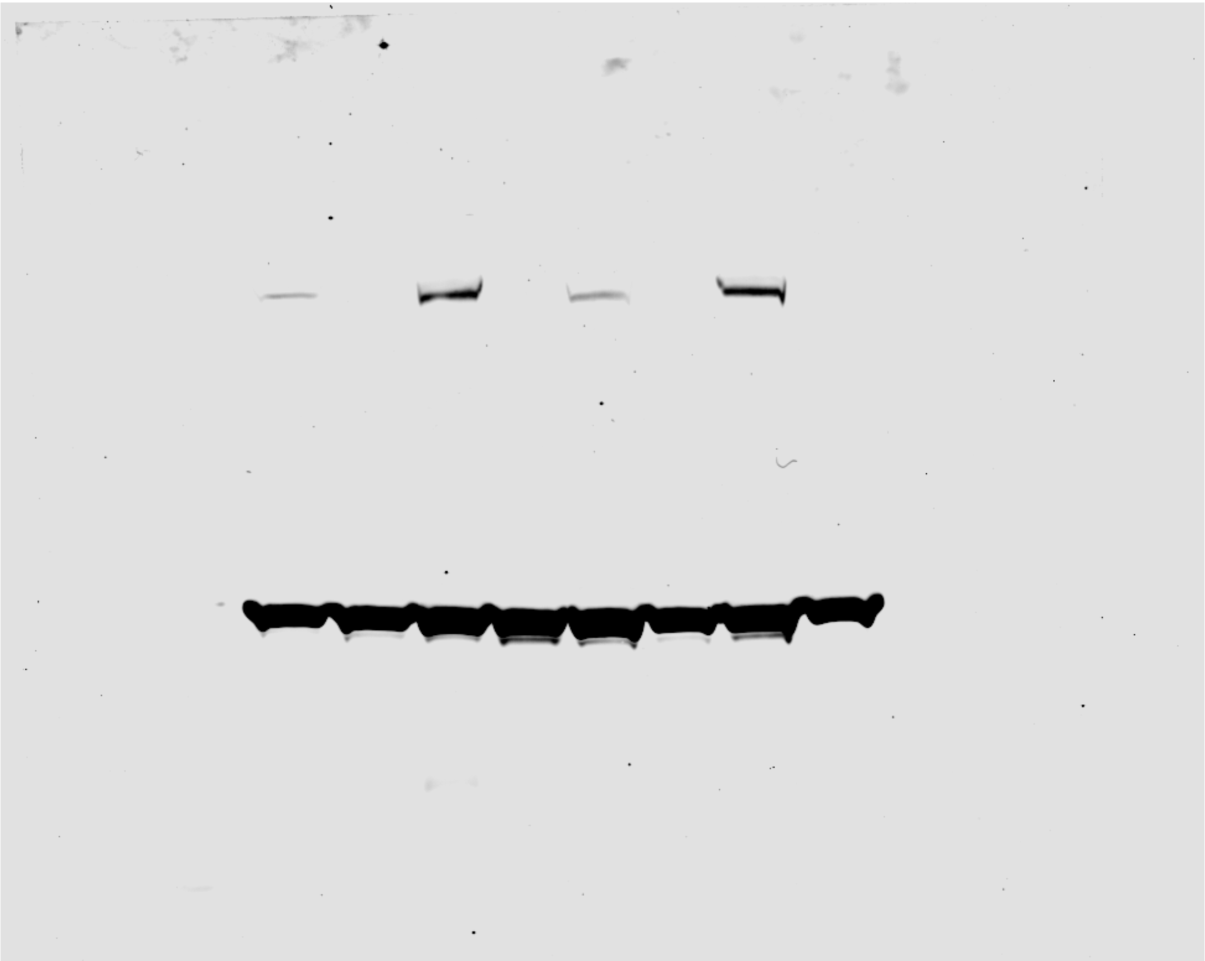

Raw western blot of NLRP3 and ACTB staining, pertaining to Supplementary Figure 5D
